# LiDAR-Based SLAM: A Review of Algorithmic Modules and LiDAR-Inertial Integration Architectures

**DOI:** 10.3390/s26196141

**Published:** 2026-09-28

**Authors:** Eslam Muhammed, Ahmed Shaker

**Affiliations:** Department of Civil Engineering, Toronto Metropolitan University, 350 Victoria St, Toronto, ON M5B 2K3, Canada; eslam.muhammed@torontomu.ca

**Keywords:** SLAM, 3D LiDAR, LiDAR-inertial odometry, sensor fusion, architectural taxonomy, state estimation, scan registration, loop closure, map representation, back-end optimization

## Abstract

**Highlights:**

**Main Findings:**

- The review presents a dual-axis, architecture-centered taxonomy of more than 100 representative LiDAR-based methods. The first axis distinguishes LiDAR-only and LiDAR-inertial sensing configurations, while the second distinguishes their principal registration, representation, coupling, and estimator structures.
- The reviewed literature shows a persistent trade-off among accuracy, computational efficiency, and robustness, where methods optimized for high precision often require greater computational resources, while lightweight real-time systems may be less robust in geometrically degraded or dynamic environments.

**Implications of the main findings:**

- Future LiDAR-based SLAM systems are likely to move beyond purely geometric matching toward modular frameworks that integrate continuous-time trajectory modeling, semantic scene understanding, adaptive mapping, and uncertainty-aware sensor fusion.
- No single SLAM architecture is universally optimal. System selection should be guided by deployment-specific factors such as platform dynamics, environmental structure, sensor configuration, computational resources, and required accuracy.

**Abstract:**

Simultaneous Localization and Mapping (SLAM) enables real-time six-degree-of-freedom (6-DOF) state estimation and spatial reconstruction in environments where global navigation satellite systems (GNSSs) are unavailable or unreliable. Light Detection and Ranging (LiDAR) is particularly suited to this task because it provides direct geometric measurements and remains largely unaffected by ambient illumination. This review presents an architecture-centered synthesis of LiDAR-based SLAM, covering LiDAR-only and LiDAR-inertial systems through a dual-axis, architecture-centered taxonomy. The first axis distinguishes sensing configuration, while the second distinguishes the principal registration, representation, coupling, and estimator structures within the corresponding branches. Core algorithmic modules, including front-end registration, state estimation, loop closure, map representation, and back-end optimization, are examined to determine how their interactions affect accuracy, computational latency, robustness, and scalability. Comparisons of representative systems are integrated within the corresponding methodological sections. Quantitative results are compared only when the same dataset subset, metric, and evaluation protocol are shared, while results obtained under non-equivalent conditions are used only as contextual evidence. The analysis identifies recurring trade-offs between low-latency recursive estimation and globally consistent graph-based optimization, as well as between sparse feature-based, direct, and learning-based representations. It also highlights the field’s progression toward continuous-time estimation, semantic scene understanding, adaptive map representations, neural implicit mapping, and uncertainty-aware sensor fusion. Finally, the review organizes future research around trustworthy estimation and recovery, environmental adaptability, persistent map maintenance, resource-bounded operation, and dependable interfaces with navigation and motion planning.

## 1. Introduction

Autonomous robotic systems are expected to navigate and operate in unknown environments without relying on pre-existing maps or external positioning aids. Simultaneous Localization and Mapping (SLAM) addresses this problem by enabling a system to estimate its pose, including position and orientation, while simultaneously constructing a map of the surrounding environment [1]. This dual capability has become fundamental across diverse domains, including autonomous driving, Unmanned Aerial Vehicles (UAVs), mobile robotics, construction automation, and agricultural applications [2,3,4].

SLAM is particularly important in environments where global navigation satellite systems (GNSSs) are unavailable or unreliable. Urban canyons, underground mines, forests, disaster-affected areas, indoor spaces, tunnels, and repetitive architectural environments often present weak GNSS signals and perceptual challenges such as poor lighting, visual ambiguity, and limited distinctive features [5,6]. In these conditions, SLAM provides autonomous systems with a means to maintain localization and environmental awareness while adapting to changing surroundings over time.

### 1.1. Sensor Modalities and the Role of LiDAR in SLAM

Although the SLAM problem can be formulated independently of a specific sensor [7], practical performance is strongly shaped by the sensing modality. SLAM systems have used cameras, including monocular, stereo, Red, Green, and Blue (RGB), and RGB-Depth (RGB-D) sensors, as well as LiDAR (2D and 3D), Radio Detection and Ranging (RADAR), and Sound Navigation and Ranging (SONAR) [8,9]. Each modality presents different trade-offs in measurement accuracy, environmental robustness, computational demand, and cost. Understanding these trade-offs is essential for selecting sensing configurations that match the target platform and operating environment.

LiDAR has become a particularly important modality for SLAM because it directly measures geometric structure. By estimating range from laser pulse travel time, LiDAR sensors generate organized three-dimensional point clouds that represent surrounding surfaces. Unlike passive optical sensors, LiDAR does not depend on ambient illumination and can operate in environments where cameras are affected by lighting changes, low texture, or visual ambiguity [9]. Monocular visual methods must also infer depth through multi-view geometry, making them vulnerable to scale ambiguity and occlusion [5]. These sensing properties make LiDAR especially suitable for SLAM in tunnels, warehouses, outdoor scenes with variable illumination, and other perceptually challenging environments.

Recent advances have further accelerated the adoption of LiDAR in robotic mapping and localization. The increasing availability of cost-effective, high-resolution LiDAR sensors, including solid-state and Micro-Electro-Mechanical System (MEMS)-based models, has reduced earlier limitations related to cost, size, and power consumption. Modern sensors are increasingly compact, energy-efficient, and better suited to mobile robotic platforms [6]. Together with LiDAR’s geometric precision, these developments have made LiDAR a central sensing modality for autonomous navigation in GNSS-denied and visually degraded environments.

However, standalone LiDAR SLAM remains limited by drift accumulation, geometric degeneracy, and motion distortion, particularly during rapid motion and in feature-sparse or repetitive environments [10]. Integrating LiDAR with other sensors helps address these limitations. More explicitly, LiDAR-inertial fusion addresses these limitations by combining high-frequency IMU measurements with LiDAR observations, supporting motion prediction, point cloud de-skewing, and short-term state estimation between LiDAR scans [11,12]. Because IMUs provide high-rate motion information but drift over time, while LiDAR provides lower-rate geometric constraints that anchor the estimate to scene structure, their integration improves robustness in challenging environments where either sensor alone may be insufficient [13]. This complementarity motivates the review’s focus on LiDAR as the primary mapping modality and on the architectural consequences of integrating inertial measurements into LiDAR-based estimation.

### 1.2. Scope, Terminology, and Taxonomic Contribution

In this review, odometry denotes local incremental trajectory estimation from sequential measurements, commonly through scan-to-scan or scan-to-local map registration. An odometry method may maintain a bounded local map, but it does not by itself jointly estimate a persistent map of an initially unknown environment. SLAM denotes joint estimation of the robot trajectory and the associated environmental map. Loop closure, relocalization, and global back-end correction determine whether accumulated drift can be corrected and global consistency maintained, while relocalization supports recovery or global alignment against an existing map. An odometry module can serve as the front-end of a SLAM system but does not, on its own, address all the other roles of SLAM. Within this LiDAR-only and LiDAR-inertial scope, the umbrella term “LiDAR-based methods” is used when the reviewed set includes odometry, registration, localization, mapping, loop-closure, or back-end components in addition to SLAM systems.

The aim of this review is to provide an architecture-centered reference that helps new readers select an appropriate LiDAR-only or LiDAR-inertial system or development baseline for a given research or deployment setting.

Existing reviews organize this topic from complementary viewpoints. Zhu et al. [5] emphasize fusion among cameras, LiDAR, and IMUs, while Zhang et al. [6] provide a broad account of the 3D LiDAR SLAM framework, challenges, taxonomy, and benchmarks. Xu et al. [9] organize multi-sensor LiDAR SLAM according to sensor composition and loose or tight coupling, and Yue et al. [14] emphasize LiDAR types and configurations for robotic mapping. Table 1 compares the scope, organizing principles, architectural level of detail, and decision-support capabilities of these surveys with those of the present review. Compared with prior surveys that emphasize multimodal sensor fusion, broad LiDAR-SLAM taxonomies, coupling strategies, or LiDAR hardware configurations, the dual-axis taxonomy adopted in this review connects sensing configuration with branch-specific architectural organization within a common decision-oriented framework. Its first axis separates LiDAR-only and LiDAR-inertial systems. The second axis uses the dominant architectural discriminator within each branch: LiDAR-only methods are organized primarily by their registration and representation paradigms, whereas LiDAR-inertial methods are organized primarily by their coupling and estimator structures.

The contribution of the present review lies in integrating individual sensing, registration, representation, coupling, and estimator categories within a focused LiDAR-only and LiDAR-inertial decision framework that consistently traces how front-end registration and representation, inertial coupling and estimator design, loop closure and relocalization, and global optimization shape computational burden, retained failure modes, correction capability, and deployment suitability. This synthesis incorporates representative developments through 2026.

Across both branches, registration, map representation, loop closure, and back-end correction are analyzed as shared architectural components. Accordingly, the LiDAR-only branch comprises feature-based, geometric direct-registration, and learning-based, semantic, and neural/implicit approaches. The LiDAR-inertial branch comprises loosely coupled, tightly coupled filter-based, and tightly coupled graph-based architectures.

The review covers more than 100 representative LiDAR-based methods and systems published between 2014 and 2026, together with earlier foundational work needed to explain core registration and estimation concepts. It includes complete SLAM pipelines and directly enables odometry, registration, localization, loop-closure, mapping, and back-end contributions. Section 4 and Section 5 provide the method-level analysis, while the appendix tables support inferred comparisons of architectural strengths, limitations, and implementation boundaries.

The review focuses on systems in which 3D LiDAR is the primary environmental sensing and mapping modality. It covers LiDAR-only methods and LiDAR-inertial integration because the IMU supplies auxiliary motion information for propagation, point cloud de-skewing, initialization, and state estimation, while the environmental map remains derived primarily from LiDAR observations. This relationship permits a consistent analysis of geometric registration, inertial coupling, estimator structure, and global correction. Although camera, GNSS, and other sensors are important extensions, their visual measurement models, calibration requirements, absolute position factors, failure modes, and evaluation protocols require separate taxonomic treatment. Therefore, they are acknowledged only when they expose an interface or extension relevant to a LiDAR or LiDAR-inertial core.

The review taxonomy, shared SLAM pipeline, and correspondence with the subsequent manuscript sections are summarized in Figure 1. LiDAR-only methods are organized by their principal registration and representation strategies, whereas LiDAR-inertial methods are organized by their integration and estimator architectures.

The remainder of the review is organized as follows: Section 2 describes the literature search, selection, classification, and comparative analysis. Section 3 introduces the technical background and SLAM pipeline concepts needed for the review. Section 4 and Section 5 examine LiDAR-only methods and LiDAR-inertial architectures, respectively, including their taxonomic synthesis and reported performance. Section 6 examines shared global-consistency functions, map scalability, and benchmark contexts. Section 7 discusses unresolved challenges and research directions, and Section 8 presents the conclusions.

## 2. Materials and Methods

This architecture-centered review used an iterative literature discovery and qualitative synthesis process. Section 2.1 describes how the literature was identified and selected, while Section 2.2 explains how the retained studies were classified and comparatively analyzed.

### 2.1. Literature Search and Selection

Literature for this architecture-centered review was assembled through iterative keyword-based searches in Google Scholar (Google LLC, Mountain View, CA, USA) and reference list searching of relevant surveys and primary technical papers. Representative search terms included “LiDAR-based SLAM,” “LiDAR SLAM,” “LiDAR-inertial SLAM,” “LiDAR-inertial odometry,” “LIO,” “loosely coupled LiDAR-inertial,” and “tightly coupled LiDAR-inertial”. Method names, estimator families, and algorithmic modules encountered during reading were subsequently used in follow-up Google Scholar searches. The review primarily covers representative works reported from 2014 to 2026, with earlier foundational studies retained where necessary to explain core registration, estimation, and SLAM concepts.

Studies were included when LiDAR was the primary environmental sensing modality and the work described either a complete SLAM system or a directly enabling odometry, registration, localization, loop-closure, mapping, or back-end contribution with sufficient technical or evaluation detail to support architectural comparison. Visual-only methods, works in which LiDAR was peripheral, non-technical summaries, duplicate or superseded versions of the same contribution, and works outside the stated architectural scope were excluded. Consistent with the sensor scope defined in Section 1.2, methods outside the LiDAR-only and LiDAR-inertial core were not treated as separate review categories. Selected multimodal systems were retained only as boundary examples of extensions to that core.

Representative works were selected to cover influential systems, distinct registration, coupling, estimator, map, or back-end architectures, implementation and benchmark evidence where available, deployment-relevant limitations and failure modes, and emerging research directions. Peer-reviewed journal and conference papers were prioritized. Recent preprints and software repositories were included selectively when they documented an emerging method or implementation not adequately represented by a peer-reviewed source, and their publication status was identified in the references.

### 2.2. Taxonomic Classification and Comparative Analysis

The reviewed methods are organized using the dual-axis taxonomy introduced in Section 1.2. Methods are compared according to sensor requirements, registration strategy, estimator design, computational demands, map representation, loop-closure and global correction capabilities, reported trajectory accuracy, and weaknesses associated with different operating conditions. Quantitative results are compared only for the reported quantity when the same dataset subset, metric, and evaluation protocol are shared. Runtime comparisons and causal conclusions about architectural superiority additionally require sufficiently aligned hardware, implementations, and experimental conditions. Results obtained under non-equivalent conditions are only used to provide context.

## 3. Technical Background and Review Framework

This section summarizes the SLAM fundamentals, LiDAR sensing and processing concepts, and coupling strategies needed to understand the systems reviewed in Section 4 and Section 5.

### 3.1. SLAM Fundamentals and Front-End/Back-End Architecture

SLAM addresses two interdependent tasks: estimating the robot’s trajectory while simultaneously constructing a consistent map of the environment from sequential sensor observations. The robot’s pose, which includes both position and orientation within a consistent reference frame, evolves over time and forms a trajectory, as shown in Figure 2. From a probabilistic standpoint, SLAM estimates the joint posterior distribution of the trajectory and the environmental map using sensor measurements and control inputs [8]. Because real-world environments often include nonlinear motion, perceptual ambiguity, dynamic objects, and non-Gaussian noise, robust SLAM requires estimation frameworks capable of maintaining consistency under uncertain and changing observation conditions [3,4,14].

In LiDAR SLAM, the front-end estimates local motion by aligning the current scan with a reference, such as the previous scan, a local submap, or a global map. This scan matching step computes the rigid-body transformation between point clouds or between extracted geometric primitives [16]. At the geometric registration level, two foundational front-end mechanisms are introduced throughout this review: feature-based methods, which extract selected geometric primitives such as edges and planes before alignment, and direct methods, which register dense or semi-dense measurements without a discrete feature-selection stage [17,18]. Learning-based, semantic, and implicit approaches are discussed separately because they may learn or augment features, correspondences, pose estimation, or map representations [19,20,21]. Projective association and continuous-time trajectory modeling are treated as specific association and motion-modeling strategies [22], while hybrid methods are classified according to their dominant registration mechanism [23,24]. Because front-end estimates are local, they inevitably accumulate drift over time.

The back-end reduces accumulated drift by incorporating additional constraints, especially loop closures. Loop closure detection identifies previously visited locations using geometric registration methods, such as Iterative Closest Point (ICP) [16], or place-recognition descriptors, such as Scan Context [25]. Validated loop closures add constraints to a pose graph, which is optimized through nonlinear least squares to improve global consistency [18]. Back-end optimization can be implemented through filtering or graph-based formulations. Filter-based methods recursively update the state while marginalizing previous states, offering computational efficiency but limited ability to re-linearize past estimates [1]. Graph-based methods preserve a history of poses and constraints, enabling global refinement at higher computational cost [18,26]. These front-end and back-end design choices provide the terminology used in Section 4 and Section 5 to compare representative LiDAR-only and LiDAR-inertial SLAM systems. Figure 3 illustrates the main components of SLAM and the relationships among sensor inputs, front-end processing, and back-end optimization.

### 3.2. LiDAR Sensors and Point-Cloud Processing

#### 3.2.1. LiDAR Sensor Characteristics

The primary input to LiDAR SLAM is a point cloud: a set of 3D coordinates (x, y, z) representing environmental surfaces, often enriched with reflectivity or intensity values. LiDAR sensors generate dense spatial data streams, often exceeding hundreds of thousands of points per second [9,14]. Key sensor parameters, including field of view (FoV), angular resolution, scan pattern, and point density, directly influence feature extraction quality, scan registration accuracy, and motion compensation requirements.

Mechanical spinning LiDAR systems, such as the Velodyne VLP-16, HDL-32E, and HDL-64E, typically provide a 360° horizontal field of view (FoV), while their vertical field of view and angular resolution depend on the number and arrangement of laser channels. Representative sensors include 16-, 32-, and 64-channel configurations, with vertical FoV values ranging from approximately 27° to 40° across common Velodyne models [27]. These sensors produce regular and repetitive scan patterns that simplify feature tracking and range-image projection, but they remain vulnerable to motion distortion and mechanical wear [27].

Solid-state LiDARs have gained popularity as a compact alternative to mechanical spinning systems [28]. Sensors such as Livox devices and the RoboSense M-series use MEMS mirrors or prism-based scanning mechanisms to reduce mechanical complexity and improve robustness. Some solid-state sensors generate non-repetitive scan patterns that densify over time within the field of view [28]. While this can increase effective coverage, it also introduces challenges, including limited range, instantaneous FoV, and non-uniform point distributions, requiring SLAM algorithms to handle irregular and sparse spatial data.

Consequently, SLAM algorithms developed around the regular ring-like scan organization of multi-beam spinning LiDARs often require adaptation for sensors with small fields of view and irregular, non-repetitive, or otherwise time-varying sampling patterns. Such adaptations can involve point organization and feature extraction, per-point motion compensation, and scan-to-map registration or data-association strategies [28]. This dependence highlights the importance of aligning SLAM algorithm design with sensor properties, since LiDAR choice directly influences downstream modules such as motion compensation, feature extraction, registration, and mapping.

#### 3.2.2. Point-Cloud Processing and Feature Extraction

LiDAR SLAM performance depends strongly on how raw point clouds are filtered, represented, and associated across time. Noise filtering removes unreliable points caused by sensor noise, multipath reflections, or environmental disturbances [29]. Common approaches include statistical outlier removal, geometric filters based on local point density, and sensor- or task-specific filtering strategies [30]. Effective filtering reduces erroneous correspondences during scan registration and improves downstream estimation stability [31].

Feature extraction identifies geometric elements in filtered point clouds that can support motion estimation, loop closure, or map construction [32]. These elements may include individual keypoints or structured primitives such as edges, planes, and surfels. The selected representation affects both reliability and computational efficiency: simple point features are inexpensive but less robust to noise, whereas planar patches and curvature-based descriptors can provide more stable constraints at higher computational cost [32,33].

Data association matches points, features, or local structures from the current scan to previous scans or to a local/global map. Approaches range from nearest-neighbor search in descriptor or Euclidean space to probabilistic models that account for uncertainty and geometric constraint-based methods that exploit structural context. Poor associations can propagate pose errors or introduce incorrect loop closures, particularly in feature-sparse or repetitive environments [33].

Together, filtering, feature extraction, and data association transform raw LiDAR measurements into geometric constraints that determine pose estimation accuracy, loop closure reliability, and real-time feasibility.

### 3.3. Limitations of LiDAR-Only SLAM

LiDAR-only SLAM systems provide accurate geometric mapping and real-time performance in many structured environments. However, reliance on a single sensing modality introduces limitations that reduce robustness in complex, dynamic, or geometrically ambiguous scenes [6,9]. A primary limitation is drift accumulation. LiDAR odometry estimates motion by registering consecutive scans or aligning scans to a local map. Small registration errors caused by sensor noise, discretization, or imperfect correspondences can accumulate over time and lead to significant trajectory divergence [4]. Loop closure can mitigate this drift by realigning the trajectory when previously visited locations are recognized, but loop detection itself becomes less reliable in repetitive or perceptually ambiguous environments.

Geometric degeneracy further limits LiDAR-only estimation when the environment does not provide enough independent constraints to observe the full 6-DoF sensor pose. Feature-based methods use geometric elements such as edges and planes to refine motion estimation [17]. In long corridors, tunnels, hallways, open fields, or other structurally repetitive environments, these features may be sparse or may constrain only selected motion directions [10]. For example, parallel tunnel walls can constrain lateral motion while providing limited information about forward translation or yaw, leading to ill-conditioned optimization and increased drift [13].

LiDAR-only systems also face temporal distortion caused by sequential scan acquisition. Mechanical LiDAR sensors typically require a finite interval, often about 100 ms, to complete a scan. During rapid or nonlinear platform motion, points within a single scan are captured from different sensor poses, producing intra-scan skew [34]. Constant-velocity motion compensation can reduce this effect, but it is less reliable during aggressive maneuvers, such as those encountered by UAVs or fast-moving robotic platforms [9]. These distortions degrade scan registration and can compound drift over time.

Together, drift accumulation, geometric degeneracy, and motion distortion indicate that LiDAR-only SLAM can be effective in structured and static environments but may lack sufficient robustness in highly dynamic, feature-sparse, or GNSS-denied settings. These limitations motivate the integration of inertial sensing, as discussed in the next subsection.

### 3.4. LiDAR-Inertial Integration and Coupling Strategies

As discussed in Section 3.3, LiDAR-only SLAM is affected by drift accumulation, geometric degeneracy, and motion distortion. LiDAR-inertial SLAM addresses these limitations by combining high-frequency IMU data, typically 100–1000 Hz, with lower-frequency LiDAR scans, typically 10–20 Hz. IMU measurements provide continuous motion information for point cloud de-skewing, scan registration initialization, and short-term state propagation, while LiDAR observations provide geometric constraints that limit inertial drift and support real-time bias estimation [9,13].

The effectiveness of LiDAR-inertial fusion depends strongly on the coupling strategy. In loosely coupled systems, LiDAR and IMU measurements are processed by separate modules, and their outputs are fused at a higher level. IMU data are commonly used for motion prediction, point cloud de-skewing, or registration initialization, but LiDAR corrections do not directly refine the IMU state or bias estimates. This modular structure is computationally efficient but limits bidirectional information exchange between sensors [9,14]. Loosely coupled systems are addressed in more detail in Section 5.1.

Tightly coupled systems incorporate raw or pre-integrated IMU measurements and LiDAR constraints within a unified state estimation framework. This formulation enables LiDAR observations to refine pose, velocity, and IMU bias estimates, improving robustness during rapid motion and in environments where LiDAR-only constraints are weak. Tightly coupled LiDAR-inertial odometry (LIO) implementations are commonly divided into filter-based approaches, such as FAST-LIO [35], which use recursive state updates for real-time efficiency, and graph-based approaches, such as LIO-SAM [13], which use factor graph optimization with IMU pre-integration to support global trajectory refinement at higher computational cost. This architectural distinction represents a central trade-off between real-time efficiency and global consistency [14]. Tightly coupled filter-based and graph-based systems are addressed in Section 5.2.1 and Section 5.2.2, respectively.

## 4. LiDAR-Only SLAM Methods and Synthesis

This section examines LiDAR-only SLAM methods that rely exclusively on LiDAR sensing, without IMU or other auxiliary sensor integration. As discussed in Section 3.3, LiDAR-only methods remain vulnerable to drift accumulation, geometric degeneracy, and motion distortion. Nevertheless, they remain important in robotic perception because of their relative simplicity, computational efficiency, and reliable performance in structured environments.

LiDAR-only methods are organized primarily by registration strategy. Feature-based methods select geometric primitives before registration, whereas direct methods align dense or semi-dense measurements without discrete feature selection. Hybrid methods are grouped by their dominant registration mechanism, while learning-based, semantic, and implicit approaches are discussed separately. These approaches differ in computational cost and robustness to noise and geometric degeneracy.

### 4.1. Feature-Based LiDAR SLAM Methods

Feature-based methods reduce computational demand by extracting sparse geometric elements from raw point clouds, supporting real-time operation. LiDAR Odometry and Mapping (LOAM) laid the groundwork for this approach by introducing an innovative dual-threaded architecture [17], which has greatly impacted the advancement of feature-based SLAM.

The high-frequency odometry thread detects edge and planar features through the assessment of local curvature. Edge features, which are typically found at sharp edges or corners, are identified by their high curvature. In contrast, planar features, which are found in flat areas, are identified by their low curvature. The odometry thread performs scan-to-scan registration by utilizing point-to-line and point-to-plane residuals. At the same time, a low-frequency mapping thread improves pose estimates by aligning the extracted features with a local map, which helps to minimize drift and enhance global consistency. Figure 4 illustrates a feature-based SLAM workflow inspired by LOAM.

Expanding upon the groundwork laid by LOAM, various enhancements improve the reliability of feature extraction. A-LOAM [36] provides a streamlined open-source reimplementation using Eigen and Ceres Solver, while retaining LOAM’s LiDAR-based curvature feature extraction, scan-to-scan odometry, and scan-to-map refinement. Other variants focus more directly on improving feature extraction. E-LOAM [37] incorporates expanded local structural information by representing the neighborhood around selected feature points with normal distributions and augments geometric features with high-intensity-variation points, enriching the sparse LOAM feature set. CSS-LOAM [38] replaces LOAM’s original feature extractor with a Curvature Scale Space (CSS)-based feature extraction strategy, evaluating curvature behavior across smoothing scales to obtain more stable feature points. Beyond enhancing LOAM’s feature extraction, some feature-based systems combine explicit primitive selection with richer map representations. Mesh-LOAM [39] is a hybrid feature-assisted point-to-mesh method that selects planar points from incoming scans and registers them against an incrementally reconstructed triangular mesh. Incremental voxel meshing and GPU-based spatial hashing support real-time operation.

Other methods refine geometric feature categorization and registration. MULLS [40] extracts and downsamples structural feature points, including ground, facade, roof, pillar, and beam classes, and performs category-constrained multi-metric ICP using point-to-plane residuals for planar features and point-to-line residuals for linear features. LiODOM [41] formulates LiDAR-only odometry as a nonlinear optimization problem based on weighted point-to-line correspondences with an adaptive local map, enabling efficient data association without relying on a full global map. GICP-LOAM [42] retains LOAM-style edge and planar feature extraction but replaces the standard feature matching stage with voxelized generalized ICP (VGICP), applying VGICP to feature-point clouds for key-to-key odometry and key-to-map registration.

LOAM has been adapted for specialized scenarios. LOAM_livox [28] adapts LOAM-style feature extraction and motion compensation to solid-state LiDARs with small FoV, irregular scan patterns, and non-repetitive sampling. M-LOAM [43] addresses multi-LiDAR odometry and mapping by estimating motion while refining inter-sensor extrinsic calibration online. LOL (Lidar-only Odometry and Localization) [44] combines LOAM-based odometry with segment-based localization against a prebuilt 3D point cloud map to reduce accumulated drift in known environments.

Some systems use LiDAR intensity as an additional feature modality. The method in [45] projects LiDAR intensity values into images and extracts ORB keypoints for scan registration. This approach is particularly useful in geometrically degenerate environments where edge and plane features may be insufficient. It combines intensity image-based feature association with 3D scan registration and map optimization, including point-to-plane constraints, demonstrating that intensity can provide complementary constraints when geometric cues are weak.

T-LOAM [46] employs Truncated Least Squares (TLS) optimization to reduce the influence of large residuals caused by outliers, sensor noise, and dynamic objects. The method extracts four geometric feature categories, namely edge, planar, spherical, and ground features, using Multi-Region Ground Extraction (MRGE) and Dynamic Curved-Voxel Clustering (DCVC). These feature residuals are then incorporated into a TLS-based pose optimization framework.

HBA [47] addresses global consistency in large-scale LiDAR mapping through Hierarchical LiDAR Bundle Adjustment. It performs bottom-up bundle adjustment within local windows to improve mapping consistency, and then applies a top-down pose graph optimization process to propagate corrections efficiently across the full trajectory. This hierarchical formulation reduces the computational burden of full bundle adjustment while preserving large-scale map consistency.

In resource-constrained and dynamic scenarios, Light-LOAM [48] uses a non-conspicuous feature selection strategy to obtain stable feature samples and introduces a two-stage correspondence selection process. The first stage uses KD-tree-based coarse matching, while the second stage applies graph-based geometric consistency voting to reject unreliable correspondences. Dynamic Object-Aware LiDAR Odometry [49] addresses urban dynamic environments by adaptively weighting feature associations from both static and dynamic objects, reducing the influence of inconsistent dynamic-object measurements without simply discarding all dynamic features.

Despite these advances, feature-based approaches remain dependent on the availability and spatial distribution of reliable geometric or appearance-based features. In structurally degenerate environments, such as long corridors or feature-sparse scenes, extracted features may fail to sufficiently constrain all 6-DoF motion, leading to ill-conditioned optimization and unobservable motion directions. This limitation motivates direct approaches (Section 4.2), which operate on denser point cloud information rather than relying only on selected features. Table A1 in the appendix summarizes the feature-based methods discussed above. Overall, the table highlights a recurring trade-off: methods that enrich feature representation or correspondence modeling, such as E-LOAM [37], MULLS [40], and Light-LOAM [48], improve robustness or accuracy under specific conditions, but often introduce additional preprocessing, matching, or optimization complexity.

### 4.2. Direct, Learning-Based, and Semantic LiDAR SLAM Methods

In contrast to feature-based methods, direct or featureless LiDAR SLAM approaches avoid explicit extraction of sparse geometric primitives and instead perform registration using dense or semi-dense point cloud information, continuous surface representations, or projected range/intensity images [50,51].

#### 4.2.1. Geometric Direct Registration Methods

Direct registration methods build on classical geometric algorithms. Iterative Closest Point (ICP) [16] estimates rigid-body transformations by iteratively minimizing Euclidean distances between corresponding points. While foundational, point-to-point ICP is sensitive to initialization, noise, and local minima. The Generalized ICP (G-ICP) [52] improves robustness by modeling local surface geometry as planes and emphasizing the minimization of point-to-plane residuals within a probabilistic framework. An alternative method is the Normal Distribution Transform (NDT) [53], which transforms environments into voxel grids, models point distributions within each voxel as multivariate Gaussians, and accomplishes registration by maximizing the observation likelihood based on the NDT framework.

Building on these fundamental techniques, a variety of sophisticated ICP adaptations have emerged to improve reliability, efficiency, and flexibility in modern SLAM systems. KISS-ICP [50] exemplifies this trend, showing that a carefully designed point-to-point ICP system can achieve competitive accuracy. The pipeline integrates constant-velocity motion prediction, scan deskewing, voxel-based subsampling, adaptive correspondence thresholding, and robust registration using a Geman-McClure loss. This demonstrates the continued effectiveness of classical ICP when the surrounding system design is carefully engineered.

MAD-ICP [54] enhances this field by introducing a mechanism for map updates that is guided by information, enabling the selective incorporation of new scans based on the pose uncertainty assessed by ICP. The approach employs a PCA-based kd-tree to efficiently represent scans and estimate surface normals, thereby enabling accurate point-to-plane registration. By aligning kd-tree leaves instead of raw points and utilizing a robust Huber loss during the optimization process, the system improves its resistance to noise, poor correspondences, and dynamic artifacts. Fast-ICP [55] interprets classical point-to-point ICP as a majorization-minimization procedure and applies Anderson acceleration to improve convergence. Its robust variant introduces a Welsch-function metric to reduce sensitivity to outliers and partial overlap.

These registration algorithms serve as front-ends for complete SLAM systems. HDL-Graph-SLAM [56] combines a modular front-end supporting both G-ICP and NDT with a graph-based back-end for loop closure and global map correction. RTAB-Map [57] can operate in LiDAR-only mode, using ICP-based scan-to-map registration with a pose-graph back-end for global consistency. LOCUS 2.0 [58] is a computationally efficient LiDAR odometry system for large-scale underground mapping. It introduces a normals-based GICP formulation to reduce covariance re-computation, an adaptive voxel-grid filter for more deterministic runtime, and a sliding-window map representation to bound memory consumption. The system was demonstrated across heterogeneous robot platforms in the DARPA Subterranean Challenge. A distinctly different approach is taken by Google Cartographer [59], which follows a different scan-matching paradigm for 2D LiDAR SLAM, aligning incoming scans against locally consistent submaps and using loop closure with pose-graph optimization to maintain global consistency. ART-SLAM [60] presents a multi-threaded architecture with real-time tracking, loop closure detection, and pose-graph optimization, enabling efficient motion tracking while supporting global trajectory correction.

Alternative systems emphasize high-performance odometry front-ends tailored to particular challenges. Direct LiDAR Odometry (DLO) [51] utilizes a G-ICP-based pipeline that is specifically optimized for environments with perceptual degradation and platforms with limited computational resources. NDT-LOAM [61] uses Normal Distributions Transform (NDT)-based registration to improve robustness in structurally ambiguous or noisy scenes and is formulated as a complete LiDAR-only solution without inertial inputs. These provide high-quality motion estimates usable directly for navigation or as inputs to separate back-end optimization modules.

Beyond discrete point correspondences, some direct methods register scans against surface-based models. SuMa [18] uses dense projective data association to align incoming scans with vertex and normal maps rendered from a surfel representation. Its surfel stability mechanism reduces the influence of transient measurements, while the same representation supports loop-closure verification and pose-graph correction. IMLS-SLAM [62] uses an Implicit Moving Least Squares (IMLS) surface representation in a scan-to-model framework, aligning incoming scans to a continuous surface model without relying on explicit feature extraction. SLAMesh [63] incrementally constructs dense triangular meshes through Gaussian Process-based surface reconstruction, substituting point-to-point registration with robust point-to-mesh alignment to enhance resilience against noise and sparsity. Puma-LO [64] utilizes Poisson surface reconstruction in local sliding windows, resulting in high-fidelity maps and precise odometry via ray-casting data association, but does not operate in real-time.

Recent developments address intra-scan motion distortion using continuous-time trajectory representations. CT-ICP [22] introduces a LiDAR-only continuous-time ICP formulation in which each scan is parameterized by poses at the beginning and end of the scan, with point positions interpolated according to their timestamps. This elastic scan-to-map registration reduces motion distortion and improves robustness under rapid or irregular motion. The method is further extended with LiDAR-only loop detection and pose-graph optimization, forming a complete SLAM system. Similarly, Traj-LO [65] utilizes piecewise linear interpolation for trajectory parameterization and optimizes the trajectory jointly with scan alignment. This approach addresses motion distortion without IMU input, making it relevant for LiDAR-only odometry under rapid motion. I2EKF-LO [66] provides another LiDAR-only approach to motion-distortion correction. It replaces IMU propagation with a uniform-motion model and uses a dual-iteration extended Kalman filter to refine motion-distortion correction during prediction and point-to-plane residuals during the measurement update. The method dynamically adjusts process noise according to confidence in the assumed motion model.

Alternative methods modify image-based direct SLAM techniques for LiDAR by utilizing 2D projections. MD-SLAM [24] builds multi-resolution pyramids utilizing LiDAR intensity or range images and executes direct photometric alignment, drawing on concepts from visual SLAM. ECTLO [67] focuses on prism-based (solid-state) LiDARs, utilizing compact 2D range images and continuous-time trajectory models to represent environments. This illustrates that transforming 3D LiDAR data into organized 2D representations can enable direct registration strategies originally developed for image-based SLAM.

SROM [68] presents a hierarchical registration approach that begins with initial alignment through Phase-Only Correlation (POC) and is subsequently refined using standard ICP, enhancing convergence when initialization is difficult.

ERASOR [69] tackles dynamic environments by employing geometry-based, learning-free preprocessing techniques to eliminate moving objects. The method assesses point consistency over several previous scans by employing a pseudo-occupancy descriptor and a “Scan Ratio Test,” effectively filtering out unstable or transient points prior to the registration process. This allows downstream registration methods, such as G-ICP and NDT-LOAM, to operate more effectively in complex, changing environments.

Table A2 in the Appendix summarizes the direct geometric methods discussed above. A recurring pattern emerges: direct methods improve robustness over feature-based approaches in unstructured or low-feature environments by bypassing explicit feature extraction, but this advantage diminishes in geometrically degenerate scenes, such as long corridors, open fields, or low-density scans, where the point distribution is insufficient to constrain all six degrees of freedom. In such conditions, both method classes are susceptible to the same fundamental limitations of single-sensor LiDAR systems such as unbounded drift accumulation, geometric degeneracy, and motion distortion.

#### 4.2.2. Learning-Based, Semantic, and Neural Methods

Although the geometric methods discussed in the previous section achieve strong performance through carefully designed registration pipelines, they remain sensitive to initialization, local minima, geometric degeneracy, and manually defined cost functions. These limitations have motivated learning-based methods that use data-driven feature extraction, learned correspondences, semantic cues, or neural map representations to improve robustness in perceptually challenging environments.

Learning-based registration methods augment conventional, explicitly designed geometric registration objectives with features learned from data. LO-Net [19] projects consecutive LiDAR scans into a structured 2D representation and uses a convolutional neural network to estimate the relative 6-DoF pose, together with auxiliary normal and mask estimation. It reports a translational error of 1.75% on the held-out KITTI odometry sequences and was trained using an NVIDIA 1080 Ti GPU. DeepVCP [70], in contrast, operates directly on 3D point clouds by learning keypoints and virtual correspondences for registration, using PointNet++-based feature extraction and a 3D CNN correspondence-generation module. It evaluates registration performance on both KITTI and Apollo-SouthBay.

PWCLO-Net [71] extends learning-based LiDAR odometry using a hierarchical pyramid, warping, and cost-volume architecture, achieving a translational error of 1.09% on KITTI sequences 07–10 and reporting experiments on an NVIDIA RTX 2080Ti GPU. More recently, ELiOT [72] adopts a transformer-based architecture to aggregate multi-scale geometric features from point clouds, reporting a translational error of 2.91% on the same held-out KITTI sequences. Its evaluation uses a high-end NVIDIA GPU with 48 GB of graphics memory. These results demonstrate the potential of learned LiDAR odometry, while also showing that reported performance is strongly tied to the training/testing split, benchmark domain, and GPU-based inference setting.

Beyond feature learning, semantic methods incorporate object or class-level information to improve robustness. SLOAM [73] uses semantic segmentation to identify stable, class-specific landmarks, such as tree trunks, in forest environments and leverages these semantic landmarks for odometry, mapping, and tree-parameter estimation. SuMa++ [20] integrates a RangeNet++ semantic segmentation front-end with the surfel-based SLAM back-end of SuMa [18]. Rather than relying only on geometry, it uses semantic labels to weight ICP residuals, improve robustness to outliers, and reduce the influence of dynamic objects such as vehicles and pedestrians. On KITTI road sequences, SuMa++ reports an average relative translational error of 1.04%, compared with 1.35% for SuMa and 1.17% for the “SuMa_nomovable” variant [20]. This improvement comes with additional computational cost: the paper reports approximately 75 ms per scan for RangeNet++ segmentation and 48 ms for surfel mapping on an Intel Xeon W-2123 CPU with an NVIDIA Quadro P4000 GPU [18,20]. INF-SLiM [74] extends semantic reasoning to large-scale implicit semantic mapping by encoding geometry and semantic information within a sparse hierarchical neural field. It performs evaluations on SemanticKITTI and SemanticPOSS datasets [75,76], which are characterized by dynamic instances. INF-SLiM reports an mIoU of 63.0% on the SemanticKITTI semantic segmentation comparison, and runs experiments on an NVIDIA GeForce RTX 4090 GPU.

Recent developments also incorporate deep learning into map representation itself. PIN-SLAM [21] employs a point-based implicit neural representation to encode scene geometry as a continuous signed-distance field. It alternates between online optimization of a local implicit map and correspondence-free scan-to-signed distance function (SDF) registration for odometry, while adding loop closure detection using polar context descriptors and pose graph optimization for global consistency. PIN-SLAM reports approximately 7.1 Hz for the full system on KITTI sequences 00–10 using a single NVIDIA Quadro A4000 GPU, with an average runtime of 0.14 s per frame. Its lighter variant reaches 11.3 Hz at a modest accuracy trade-off [21]. Unlike offline supervised pose-regression methods, PIN-SLAM does not require task-specific pre-training, because its neural representation is optimized online from the input scene. In contrast, SHINE-Mapping [77] is a mapping-only method that assumes known poses. It adopts a sparse hierarchical implicit neural structure for large-scale mapping and reports strong reconstruction accuracy on MaiCity and Newer College, including lower Chamfer-L1 error than the compared reconstruction baselines on both datasets.

LocNDF [78] formulates the map as a neural distance field and uses it for scan registration and Monte Carlo localization. Its experiments are reported on an Intel i7 CPU with an NVIDIA RTX A5000 GPU, and the Monte Carlo localization experiment runs at approximately 2.6 Hz. LONER [79] prioritizes online operation for neural implicit LiDAR SLAM, reporting an average tracking latency of 14 ms and an online map update rate of approximately 18 Hz on the Fusion Portable and Newer College datasets, using an AMD Ryzen 5950X CPU and an NVIDIA A6000 GPU.

Across these implicit neural systems, the main advantage is that geometry is represented as a continuous field rather than only as an explicit point cloud, surfel map, or voxel occupancy grid [21,79]. However, it would be inaccurate to say that they completely eliminate discrete structures: practical implementations often still use sparse neural points, octrees, grids, or other spatial indexing mechanisms for efficiency [74,78]. Reported real-time or online performance also generally relies on GPU acceleration, which remains an important deployment constraint for resource-limited robotic platforms [74,79].

While learning-based and semantic methods offer clear advantages for perceptual ambiguity, dynamic environments, and compact map representation [19,79], they also introduce practical challenges. Supervised methods require large datasets with accurate labels or trajectories, and models trained on one distribution may generalize poorly to substantially different environments [19,71]. Neural implicit methods reduce some dependence on offline supervised pose training, especially when optimized online, but they still introduce additional optimization cost and hardware requirements [74,79]. Therefore, learning-based methods are best viewed as complements to geometric SLAM rather than direct replacements. Hybrid systems that combine geometric priors, semantic cues, and learned representations appear especially promising for improving robustness while preserving interpretability and real-time performance.

Table A3 in the appendix summarizes representative learning-based and neural implicit LiDAR SLAM methods. The supervised KITTI-trained methods reviewed here show measurable dependence on the training/testing split: LO-Net [19] reports 1.75% translational error on held-out KITTI sequences, PWCLO-Net [71] reports 1.09% on KITTI sequences 07–10, and ELiOT [72] reports 2.91% on the same held-out sequences. By contrast, PIN-LO, the odometry-only variant of PIN-SLAM [21], reports 0.51% on KITTI sequences 00–10 and 0.64% on the KITTI leaderboard sequences 11–21 without offline pre-training. Although these values were reported under different training and evaluation settings, they are included as contextual evidence rather than as a numerical ranking. Nevertheless, they illustrate an architectural distinction: LO-Net, PWCLO-Net, and ELiOT rely on offline supervised training, whereas PIN-LO optimizes its representation online without specific pre-training.

Even with progress in LiDAR-only SLAM via geometric and learning-based approaches, the core challenges highlighted in Section 3.3, such as drift accumulation, geometric degeneracy, and motion distortion, are not eliminated.

### 4.3. Taxonomic Synthesis of Scan Registration Paradigms

Table 2 synthesizes the preceding method-level descriptions according to their primary registration mechanism: explicit geometric feature selection, direct geometric alignment, and learning-based or implicit neural estimation. The comparison identifies where front-end computation is concentrated, the geometric or operational conditions under which each paradigm may fail, and the map representations used for registration. Individual methods are summarized in Appendix Table A1, Table A2 and Table A3. The taxonomy describes recurring architectural trade-offs rather than a universal performance ranking, because accuracy and runtime remain dependent on implementation, dataset, sensor configuration, and experimental protocol.

#### Comparative Analysis of Front-End Registration Methods

Feature-based front-ends such as LOAM [17] can reduce registration cost by operating on selected edge and planar primitives rather than the complete scan. As detailed in Section 4.1, this supports real-time operation but depends on reliable and spatially distributed features. Environments such as tunnels, highways, open fields, and long corridors can leave one or more motion directions weakly constrained.

Direct registration methods reduce dependence on handcrafted features by using dense or semi-dense point cloud information. This can improve robustness when explicit feature extraction is unreliable, but shifts computation toward correspondence search, local-map maintenance, residual construction, and filtering. Efficient data structures and simplified registration are therefore central to real-time operation. DLO [51] demonstrates the influence of map organization through adaptive keyframing, a local submap, and NanoGICP registration: in its study, processing decreased from 74.2 ms per scan with radius-based sub-mapping to 21.6 and 19.1 ms with its two keyframe schemes on a four-core Intel i7 CPU. The KISS-ICP pipeline described in Section 4.2.1 also maintains competitive accuracy across KITTI [80], MulRan [81], Newer College [82], and NCLT [83] through its compact direct-registration design [50]. These results show that direct-registration latency depends not only on the residual formulation but also on data association, downsampling, map updating, keyframing, and parameter design.

Continuous-time registration addresses motion distortion by estimating an intra-scan trajectory rather than assigning a single rigid pose to the complete scan. CT-ICP [22], described in Section 4.2.1, implements this principle through continuous-time trajectory estimation combined with direct point cloud registration. This can improve robustness under rapid motion and significant intra-scan distortion, but introduces additional trajectory states and optimization complexity.

Hybrid strategies extend this trend by adding appearance or semantic cues when geometry alone becomes weak, repetitive, dynamic, or ambiguous. For example, methods utilizing LiDAR intensity [45] project return values into images to track photometric features in structurally degenerate environments, while semantic frameworks like SuMa++ [20] incorporate deep-learning-based segmentation labels to filter dynamic objects and weight ICP residuals. Overall, feature-based methods prioritize efficiency but depend on stable geometric primitives. Direct methods broaden usable geometric evidence but require efficient correspondence search and map maintenance. Continuous-time methods improve motion-distortion handling but increase optimization complexity. Hybrid or adaptive methods add complementary cues or adjust map resolution at the cost of additional sensing, calibration, training, or computation.

This architectural trade-off is reflected in Figure 5, which compares selected LiDAR-only methods using the common KITTI odometry leaderboard metric and test sequences 11–21 [80]. Feature-based LOAM achieves an average relative translational error ($t_{\mathrm{rel}}$) of 0.55%, while the continuous-time direct-registration method CT-ICP and the discrete direct-registration method KISS-ICP achieve 0.59% and 0.61%, respectively [17,22,50,80]. Structural-class feature models like MULLS (0.65%) and online implicit neural fields like PIN-SLAM (0.64%) similarly converge within this margin [21,40,80]. Five of the six selected methods therefore occupy a narrow accuracy range despite using different registration paradigms. SuMa’s higher error demonstrates that this convergence is not universal across all representations and implementations.

Similar KITTI accuracy does not imply equivalent computational behavior. The corresponding leaderboard values, method categories, and reported runtime conditions are compiled in Appendix Table A7, Group A. Most selected geometric methods use CPU-oriented implementations, whereas the reported PIN-SLAM entry relies on GPU-supported online optimization of an implicit neural map [21,80]. Because the hardware and runtime conditions were not standardized, Figure 5 supports comparison of trajectory error but not computational efficiency. Geometric methods concentrate computation on point cloud downsampling, correspondence search, residual construction, and local-map access, whereas PIN-SLAM additionally optimizes and queries an implicit neural representation online. Comparable leaderboard accuracy can therefore conceal substantially different latency, hardware, and system-integration requirements.

Regardless of registration paradigm, LiDAR-only estimation remains dependent on the availability and observability of scene geometry. Independent inertial constraints can improve motion estimation, de-skewing, and state observability in geometrically weak or rapidly changing conditions, motivating the LiDAR-inertial architectures examined in Section 5.

## 5. LiDAR-Inertial SLAM Architectures and Synthesis

Building on the coupling strategies introduced in Section 3.4, this section reviews loosely coupled fusion (Section 5.1), tightly coupled filter-based fusion (Section 5.2.1), and tightly coupled graph-based fusion (Section 5.2.2), focusing on their integration strategies, performance trade-offs, and representative systems.

### 5.1. Loosely Coupled LiDAR-Inertial Integration

In loosely coupled architectures, LiDAR and IMU measurements are not jointly optimized within a single estimator [5,6]. Instead, inertial information is typically used to support LiDAR processing, for example, by providing an initial motion estimate or assisting point cloud motion compensation. This design usually preserves a modular LiDAR odometry pipeline, but it limits feedback from LiDAR optimization to the inertial state. As a result, IMU bias estimation is not handled as part of the LiDAR optimization, and long-term consistency remains primarily dependent on the LiDAR front-end and any separate loop-closure module. Figure 6 illustrates this workflow.

Several influential systems follow this modular design. Building on LOAM, LeGO-LOAM [84] enhances the architecture of LOAM by incorporating IMU data for ground segmentation and motion initialization. Estimates of roll and pitch obtained from IMU data aid in the extraction of the ground plane in 2D range images, whereas position and velocity estimates from straightforward integration are instrumental in initializing a two-stage Levenberg–Marquardt optimization. Nonetheless, the optimization does not incorporate IMU states and biases, thereby preserving the loosely coupled approach. Several later adaptations build on this modular structure.

Scan Context [25] was integrated with LeGO-LOAM [84] to improve loop closure detection by providing a rotation-tolerant global LiDAR descriptor for place recognition. In such variants, the front-end remains largely LeGO-LOAM-like, while Scan Context strengthens global consistency through improved loop detection rather than through tighter LiDAR-IMU state estimation. Lee et al. [85] proposed Adaptive LeGO-LOAM (ALeGO-LOAM), which replaces LeGO-LOAM’s fixed sampling and feature extraction approaches with range-adaptive processing. The method divides the point cloud into short-, medium-, and long-range groups, applies different cluster size and edge/plane roughness thresholds to each group, and limits the number of processed points to maintain real-time operation. This approach preserves potentially useful distant points that fixed thresholds may reject while controlling computational load. Because these modifications affect LiDAR preprocessing rather than LiDAR-IMU state estimation, the system retains the loosely coupled architecture of LeGO-LOAM. Adaptive-LIO [86] extends the loosely coupled category by introducing an adaptive failure-aware LiDAR-inertial odometry framework. The method is explicitly described as a loosely coupled Adaptive-LIO system. It switches between LiDAR-inertial odometry and LiDAR-only odometry modes when IMU saturation or faults are detected, rather than switching between loosely and tightly coupled fusion. It also uses observability-aware adaptive frame segmentation and multi-resolution voxel maps to adjust processing according to scene structure and spatial scale.

LOFF [87] provides a related loosely coupled multimodal extension for degeneracy handling. Its LiDAR SLAM and optical-flow odometry pipelines operate independently. When LiDAR degeneracy is detected, LOFF replaces the unreliable directional component of the LiDAR estimate with optical-flow odometry, rather than combining the measurements within a unified filter or graph.

These systems illustrate the advantages and limitations of loosely coupled fusion. Their modular structure makes them computationally efficient and suitable for real-time operation, and adaptive variants such as Adaptive-LIO further improve robustness by handling IMU saturation and scene-scale variation. However, because inertial information is not jointly optimized with LiDAR measurements in a unified estimator, IMU bias correction and bidirectional sensor feedback remain limited. These limitations motivate tightly coupled methods that jointly estimate LiDAR and inertial states within unified filtering or graph-optimization frameworks. Table A4 summarizes representative loosely coupled LiDAR-inertial systems.

### 5.2. Tightly Coupled LiDAR-Inertial Integration

Tightly coupled fusion formulates state estimation as a unified probabilistic optimization problem incorporating LiDAR-derived geometric residuals and inertial measurements, enabling bidirectional correction of IMU biases and LiDAR drift [9]. Two main implementations are examined here: filter-based fusion (Section 5.2.1) and graph-based optimization (Section 5.2.2), each presenting distinct computational trade-offs between real-time efficiency and global consistency. Figure 7 illustrates a general workflow for tightly coupled approaches.

#### 5.2.1. Filter-Based Tightly Coupled Integration

Filter-based tightly coupled systems employ variants of the Kalman filter in a prediction-update cycle. High-frequency IMU measurements continuously propagate the state, while LiDAR-derived geometric constraints subsequently update it. Within the tightly coupled estimator, both measurement streams jointly refine all state components, including time-varying accelerometer and gyroscope biases. This integrated estimation process improves robustness under aggressive motion, in dynamic environments, and under geometrically sparse conditions.

The most influential filter-based systems are the FAST-LIO series, which demonstrate that careful algorithmic engineering enables real-time processing of thousands of raw LiDAR points. FAST-LIO [35] introduced a groundbreaking approach to Iterated Extended Kalman Filtering (IEKF) by presenting an innovative Kalman gain formulation. This advancement significantly decreased computational complexity, shifting it from being dependent on the number of measurements to being based on a fixed state dimension. This facilitated the immediate integration of numerous LiDAR feature points, including edges and planes, alongside IMU data. FAST-LIO2 [88] eliminated the necessity for feature extraction by directly aligning raw LiDAR points with a progressively updated map, facilitated by a novel incremental k-d tree (ikd-tree) data structure. This clear approach enhanced both adaptability and accuracy, particularly in feature-scarce environments, establishing FAST-LIO2 as a prominent standard for LiDAR-inertial odometry. The Faster-LIO [89] substitutes the traditional tree-based structure by using sparse incremental voxels and voxel hashing to accelerate nearest-neighbor search and map updates, avoiding the repeated maintenance costs of tree-based structures. This enhancement significantly lowered memory usage and matching delays, rendering the system exceptionally well-suited for extensive or evolving environments.

In addition to the FAST-LIO family, recent research explores essential constraints of conventional IEKF formulations by employing different mathematical approaches. The Eq-LIO [90] models the navigation state on ${SE}_{2}\left(3\right)$ and uses semidirect-product symmetry to incorporate IMU biases and LiDAR-IMU extrinsic parameters within an equivariant filter. Its error dynamics are linearized at a fixed origin, reducing linearization errors, which would usually lead to divergence in standard IEKF, and improving estimator consistency and robustness during abrupt state changes and aggressive motion. Gravity is represented as a fixed-magnitude vector whose two-degree-of-freedom direction is estimated, avoiding redundant gravity parameters. The method also estimates the LiDAR-IMU extrinsic rotation and translation, providing self-calibration online. Point-LIO [91] introduces a fundamentally different architecture designed for extremely aggressive motion: a point-by-point update framework that processes each LiDAR point individually at its precise sampling time, eliminating in-frame motion distortion and enabling extremely high-frequency odometry output (4–8 kHz). The kinematic model, enhanced by a stochastic process, considers IMU measurements as outputs of the system instead of inputs. This approach enables ongoing motion estimation, even in situations where the IMU experiences saturation.

SE(3)-LIO [92] approaches state propagation and updates directly on the SE(3) manifold by utilizing exponential and logarithmic maps, considering the complete 6-DOF pose as a unified geometric entity. This results in a more seamless propagation of uncertainty and effectively tackles the notable interpolation errors that occur in discrete time within highly dynamic settings. MSC-LIO [93] modifies the Multi-State Constraint Kalman Filter (MSCKF) framework for LiDAR-inertial odometry by incorporating LiDAR same-plane cluster (LSPC) tracking. In contrast to conventional methods that focus on explicit plane extraction, MSC-LIO enhances frame-to-frame data association by monitoring identical-plane observations across several keyframes. This approach removes the requirement for null-space projection and significantly boosts processing speed by almost threefold. It also introduces a novel point-velocity-based approach to accurately estimate the LiDAR-IMU time delay online.

Efficient map representations are critical for real-time filter-based SLAM. The VoxelMap series illustrates the continuing development of voxel-based mapping. VoxelMap [94] presented a novel approach with probabilistic planar voxels that capture both occupancy and surface geometry, facilitating swift nearest-neighbor queries and enhancing data association, especially in low-texture settings. VoxelMap++ [95] improves this method by integrating mergeable voxel representations and plane merging techniques through union-find algorithms, which successfully minimizes spatial redundancy and supports incremental covariance estimation within an IEKF framework. The system supports both LiDAR-only and LiDAR-inertial configurations and targets embedded and resource-constrained platforms. R-VoxelMap [96] introduces an innovative method utilizing recursive plane-fitting, wherein each voxel is integrated with a locally fitted plane model along with residual statistics. The system employs a geometry-driven recursive method for updates, utilizing RANSAC to fit planes and successfully identify outliers. Following this, it extends these outliers to deeper octree levels for additional recursive analysis. This method for identifying and reusing outliers ensures a thorough representation of the surroundings while maintaining potentially useful information. GenZ-LIO [97] tackles the challenge of the indoor–outdoor boundary by dynamically adjusting voxel resolution using a proportional-derivative (PD) feedback controller, ensuring a consistent information density during indoor–outdoor scale transitions. The hybrid-metric update effectively combines point-to-plane and point-to-point residuals within an error-state iterated Kalman filter (ESIKF) framework, addressing LiDAR degeneracy in featureless corridors. The voxel-pruned correspondence search effectively minimizes computational demands without compromising accuracy. GenZ-LIO shows reliable performance without the need for dataset-specific adjustments and introduces the NarrowWide dataset for assessing extreme transitions between indoor and outdoor environments. iG-LIO [98] combines generalized ICP with IMU in the context of MAP estimation. This approach effectively removes the need for kd-tree searches by utilizing voxel-based surface covariance estimation along with an incremental voxel map.

In addition to voxel-based methods, various systems aim to improve geometric modeling and quantify uncertainty more effectively. LOG-LIO [99] presents Ring FALS (ring-based fast approximate least squares), which is a method for directly estimating accurate surface normals from the LiDAR ring structure. This approach integrates hierarchical data association that emphasizes point-to-surfel matching within an IEKF framework. LOG-LIO2 [100] enhances this by incorporating detailed per-point uncertainty modeling, which considers both inherent sensor noise and external environmental influences such as surface roughness and incident angle. It presents LUFA (Local Uncertainty Fast Approximation), which employs incremental Jacobian matrices to efficiently propagate uncertainties in constant time, thereby enabling the IEKF to more precisely weight residuals. LIO-PPF [101] employs a plane pre-fitting strategy that incrementally tracks basic skeleton planes through plane pre-fitting, IMU-assisted initialization, and iterative PCA, reducing the cost of repeated kNN-based plane fitting. LIO-GVM [102] conceptualizes LiDAR scans and maps as Gaussian distributions utilizing a Gaussian voxel map (GVM). This method enables multi-resolution mapping, probabilistic scan matching, and adaptive voxel sizing, guaranteeing robust performance in challenging, noisy conditions. Plane-to-plane LiDAR-inertial odometry (PPLIO) [103] employs a two-step extraction method that incorporates geometric curvature analysis and eigenvalue decomposition within the Multi-State Constraint Kalman Filter (MSCKF) framework, followed by RANSAC for outlier rejection, thereby improving robustness in scenarios of insufficient initialization. COIN-LIO [104] utilizes LiDAR intensity in a tightly integrated iEKF framework, projecting intensity returns into images while employing innovative filtering techniques for brightness consistency and strategically choosing patches that offer insights in geometrically ambiguous directions.

Several systems employ alternative Kalman filter formulations or target specific operational constraints. LINS [105] uses an Iterated Error-State Kalman Filter (IESKF) with robust outlier rejection for urban navigation, and its modular design facilitates the integration of additional sensors. The open-source ESKF_LIO [106], derived from FAST-LIO, performs tightly coupled LiDAR-inertial estimation using an iterated ESKF. It employs point-to-plane scan-to-map residuals and an ikd-Tree local map. Its preprocessing was derived from LIO-SAM, and it supports feature-based and feature-disabled operation. It also supports several spinning and solid-state LiDAR models and multi-LiDAR input. Inv-LIO [107] utilizes an Invariant EKF that harnesses Lie group properties to ensure geometric consistency and minimize linearization errors in the context of aggressive motion. SW-LIO [108] creates a direct connection between the current state and earlier keyframes within a sliding window, improved by innovative ground, bias, and gravity residuals in its ESKF for enhanced initial values. LIO-EKF [109] examines traditional EKF fusion by introducing an adaptive data-association threshold that considers pose uncertainty, voxel discretization, and measurement noise. This leads to similar accuracy while notably decreasing computational demands and the need for parameter tuning. SR-LIO [110] retains an iEKF-based estimator while addressing temporal sparsity in spinning LiDARs through sweep segmentation and virtual sweep reconstruction, producing higher-frequency pseudo-scans for more frequent state updates. The framework in [111] introduces a localization method based on the Square Root Inverse Filter (SRIF), aimed at achieving numerical stability on resource-limited platforms. However, it is specifically tailored for localization with respect to prior maps, rather than for comprehensive Simultaneous Localization and Mapping.

Alternative systems tackle challenges that are specific to individual sensors. FMCW-LIO [112] utilizes Doppler velocity measurements obtained from Frequency-Modulated Continuous Wave LiDARs. It employs an on-manifold IESEKF to integrate Doppler and IMU data, enabling velocity estimation without the need for correspondences in environments that lack structural features. RI-LIO [113] enhances the IEKF approach by incorporating reflectivity-based alignment, which introduces photometric residual terms and refined LiDAR projection models to improve performance in environments characterized by dense vegetation or low feature visibility. EKF-LOAM [114] is a closely integrated enhancement of LeGO-LOAM, which combines LiDAR, IMU, and wheel odometry within an EKF framework to enhance drift resistance in geometrically constrained settings like tunnels.

DLIO [115] is included here as an observer-based exception to the predominantly Kalman filter formulations discussed in this subsection. Rather than using an IEKF/ESKF as its state estimator, it constructs a coarse inter-sweep trajectory through discrete IMU integration and evaluates analytical continuous-time equations for parallel point-wise deskewing. Motion correction and the GICP registration prior are constructed within the same processing step before direct scan-to-map registration. The resulting registration pose is used as the correction signal for a hierarchical nonlinear geometric observer, which recursively estimates pose, velocity, and accelerometer and gyroscope biases. These estimates initialize the subsequent deskewing and registration cycle. SLICT2 [116] is a second non-Kalman exception retained here because of its real-time local LiDAR-inertial estimation role. It represents the trajectory using B-splines but replaces conventional general-purpose nonlinear optimization with a customized Eigen-based linear solver and a “solve-associate-solve” update schedule. This formulation supports dense control points and repeated feature association while maintaining real-time operation under highly dynamic motion. Unlike DLIO’s recursive geometric observer, SLICT2 estimates the continuous-time trajectory by solving for spline parameters and is therefore more optimization-oriented.

Hybrid systems integrate the real-time efficiency of filtering with the global consistency of optimization-based SLAM. HD-LIO [117] is a hierarchical LiDAR-inertial odometry that addresses distribution covariance degeneration through a two-level architecture. The low level uses an IEKF with anti-degeneration point-to-distribution constraints to provide fast state estimates. The high level refines these estimates via a sliding-window factor graph incorporating IMU pre-integration factors and LiDAR observation constraints. This decoupled architecture facilitates agile local estimation and global refinement, proving especially beneficial in extended missions where accumulated drift needs correction while maintaining runtime efficiency.

The highway SLAM system [118] serves as a filter-based solution for dynamic environments. It employs CNN-based segmentation to detect and filter moving objects, subsequently providing a static point cloud to a tightly coupled ESKF for high-frequency odometry. Additionally, it conducts lower-frequency NDT-based frame-to-model registration to achieve globally consistent mapping.

Adaptive systems modify their estimation strategies in response to varying environmental conditions and the reliability of sensors. DAMS-LIO [119] introduces a degeneration-aware sensor fusion approach that identifies when LiDAR registration is in a degenerate state and seamlessly transitions to integrate relative pose information from external odometry sources, such as visual or wheel data, as direct measurements during the filter update process. The theoretical analysis grounded in the Cramer-Rao Lower Bound demonstrates that this measurement-based fusion strategy provides a more precise estimation compared to earlier methods. Chen et al. [120] present a calibration and odometry framework for UAVs equipped with actuated LiDAR systems. Its LM-Calibr component performs targetless LiDAR-motor calibration using Denavit-Hartenberg modeling, while EVA-LIO adapts the odometry configuration to environmental conditions. EVA-LIO uses a base-frame IMU rather than an IMU mounted on the rotating motor frame, reducing the risk of saturation under combined platform and actuator motion. Beyond actuated systems, calibration-aware methods also estimate time-varying extrinsic parameters in spinning-LiDAR configurations. OMC-SLIO [121] integrates extrinsic calibration parameters directly into the state vector. This approach facilitates the online calibration of spinning LiDAR platforms, effectively addressing calibration changes associated with rotating mechanisms, including installation deviations, rotational wear, and thermal effects.

Recent advancements delve into learning-driven map representations and neural implicit modeling. KN-LIO [122] integrates an EKF with a neural implicit Signed Distance Function (SDF) mapping module, enabling continuous surface reconstruction without the need for voxel discretization. This facilitates the creation of more refined and detailed maps via online neural network updates that adjust to incoming LiDAR data, paving the way for dense, high-fidelity mapping with learning-based adaptability. A complementary line of extension integrates a third sensing modality into the filter-based LiDAR-inertial framework. R3LIVE [123] couples a LiDAR-inertial odometry subsystem with a concurrent visual-inertial odometry subsystem through a shared error-state iterated Kalman filter, generating dense real-time RGB-colored 3D maps. The VIO subsystem minimizes frame-to-map photometric error to render map texture, while the LIO subsystem maintains the geometric structure, both operating within the same ESIKF estimator without switching strategies or mode selection.

The progression from feature-dependent filter-based systems such as FAST-LIO [35] to direct registration in FAST-LIO2 [88], voxel-accelerated variants such as Faster-LIO [89] and iG-LIO [98], and specialized extensions for degeneracy, reflectivity, Doppler sensing, calibration, and neural mapping shows a clear trend toward more adaptive and sensor-aware LiDAR-inertial odometry. However, these systems differ substantially in their assumptions, sensor requirements, and map representations, so their strengths are constrained by their intended operating conditions. This progression reflects a broader effort to reduce preprocessing overhead, improve robustness in feature-sparse or degenerate environments, and adapt filtering-based LIO to increasingly diverse sensing conditions. Table A5 in the appendix summarizes and compares representative filter-based tightly coupled systems.

#### 5.2.2. Graph-Based Tightly Coupled Integration

Tightly coupled graph-based SLAM frameworks formulate state estimation as nonlinear optimization over factor graphs. Each node represents the robot’s state (pose, velocity, and IMU biases) at specific time instances, while edges encode constraints from sensor observations, LiDAR scan alignments, and IMU pre-integrations. This approach facilitates adaptable integration of multiple sensors, allows for deferred optimization in loop closures, and ensures coherent global mapping. The process of IMU pre-integration produces relative motion factors among keyframes, facilitating high-frequency inertial updates while maintaining computational efficiency.

The LIO-SAM framework [13] stands out as a pivotal development in this field, effectively combining IMU pre-integration, LiDAR odometry, and loop closure detection into a cohesive factor graph that is optimized using incremental smoothing and mapping (iSAM2) from the Georgia Tech Smoothing and Mapping (GTSAM) library. The system achieves real-time state estimation through a LiDAR-inertial front-end, while maintaining global consistency via back-end optimization that incorporates loop closure constraints. This modular design allows for the straightforward integration of additional measurements and constraints, such as GPS and loop-closure constraints, thus improving adaptability across a range of operational environments. LIO-SAM has become the benchmark for graph-based LiDAR-inertial SLAM, which has inspired a range of extensions and adaptations.

Several systems build on LIO-SAM’s optimization architecture or modify its front-end to meet particular operational requirements. MILIOM [124] advances the framework to accommodate multi-input LiDAR setups by utilizing a time-synchronized front-end that integrates feature clouds from various LiDARs with complementary fields of view. The unified representation enables robust, low-drift estimation in scenarios (e.g., aerial vehicles) where single LiDAR coverage is insufficient. LIO-mapping (LIOM) [125] utilizes sliding window optimization that integrates LiDAR and IMU measurements, employing a feature-based front-end along with scan-to-map residuals. DLIOM [126] replaces sparse handcrafted feature extraction with direct scan-to-submap registration using probabilistic local map representations. This provides denser geometric constraints and improves robustness in scenes where edge and planar features are sparse or unstable.

DMSA-SLAM [127] takes a direct approach to comprehensive multi-scan optimization and adds Dense Multi Scan Adjustment (DMSA), which processes point clouds densely without needing to extract features explicitly. DMSA combines several point clouds into a single global representation instead of using edges or planes like LIO-SAM [13] or LIOM [125]. It then represents the contents of these clouds as multivariate normal distributions within uniform grid cells. This formulation means that non-matching points from dynamic objects are automatically down-weighted through the normal distribution fitting, eliminating the need for an explicit outlier rejection mechanism. The multi-scan residuals are added to a tightly coupled factor graph with IMU preintegration factors and optimized with Levenberg–Marquardt. This supports both sliding window odometry and large-scale keyframe optimization. DMSA-SLAM improves localization and mapping accuracy relative to feature-based baselines in the evaluated scenarios, particularly when scan overlap is limited. However, this accuracy comes at a computational cost, with reported processing times approximately 1.5 to 4 times longer than recording time, with Jacobian computation identified as the main bottleneck.

Recent studies combine visual data within graph-based structures to take advantage of the strengths of different sensors. PA-LVIO [128] presents a cohesive method for LiDAR-visual-inertial odometry, merging point-based LiDAR features, visual feature tracks, and IMU pre-integration into a unified factor graph. The primary innovation lies in pose-only bundle adjustment, which eliminates the need for maintaining landmark states, thereby greatly decreasing computational complexity. Frame-to-frame measurements are expressed as multi-state constraints (MSCs), offering direct relative pose constraints, whereas frame-to-map LiDAR constraints effectively mitigate drift. The system delivers real-time performance across both desktop and ARM platforms, successfully producing RGB-rendered point cloud maps.

To address the difficulties of consistency, an enhanced bundle adjustment method is designed to mitigate estimator inconsistency due to insufficient feature parameterization [129]. This framework employs stereographic projection for feature parameterization, thereby enabling First Estimate Jacobian (FEJ) techniques to uphold estimator consistency and ensure numerical stability throughout the Levenberg–Marquardt optimization process. Through the formulation of LiDAR-inertial bundle adjustment on appropriate manifolds with constraints that preserve observability, the system demonstrates enhanced consistency relative to traditional methods.

Various systems improve observability by incorporating extra sensing modalities. LVI-SAM [130] extends the smoothing-and-mapping framework by coupling visual-inertial and LiDAR-inertial subsystems. Visual odometry constraints, LiDAR odometry constraints, IMU preintegration, and loop-closure factors are incorporated to improve robustness when either visual or LiDAR sensing becomes degraded. LIO-Fusion [131] integrates GNSS, relocalization, and wheel odometry, featuring a health-checking module that verifies sensor inputs prior to factor graph integration, resulting in significant resilience to individual sensor failures.

Various systems tackle the distinct challenges posed by contemporary sensor platforms. For example, LiLi-OM [132] enhances tightly coupled methods for solid-state LiDARs (Livox Horizon) by introducing innovative feature extraction designed for irregular, non-repetitive scan patterns, while preserving a lightweight feature-based front-end alongside hierarchical keyframe-based sliding window optimization.

Additional specialized systems encompass R-LIO [133], which utilizes multi-stage registration (3D NDT front-end, line/surface feature back-end) along with nonlinear motion distortion compensation, and Spin-LOAM [134], which presents adaptive scan accumulation that evaluates spatial feature distribution to optimize the balance between matching frequency and accuracy.

An increasing number of tightly coupled graph-based systems utilize continuous-time trajectory representations to effectively manage motion dynamics and asynchronous sensing. CLINS [135] employs B-splines to model platform trajectory, achieving effective LiDAR-IMU fusion through tightly coupled methods and utilizing “non-rigid registration” to eliminate motion distortion. The two-stage loop closure correction executes discrete pose-graph optimization on keyframes, subsequently aligning B-spline trajectories to the optimized poses. This approach effectively mitigates drift while maintaining the integrity of the local trajectory shape.

Wildcat SLAM [136] introduces a strong framework featuring a sliding-window odometry front-end that employs B-spline interpolation to create “surfel” map primitives, along with a pose-graph optimization back-end. The primary innovation lies in its scalability, achieved through the use of “submaps”, collections of local odometry and surfel data that serve as essential graph nodes. This approach facilitates efficient multi-agent mapping by allowing robots to share submaps with one another. IN2LAAMA [137] investigates the upper limits of accuracy in offline batch optimization, employing Gaussian Process regression to produce Upsampled Pre-integrated Measurements (UPMs) from raw IMU data. This method of probabilistic interpolation facilitates precise motion characterization at any given timestamp, allowing for accurate correction of motion distortion through the joint optimization of poses, IMU biases, time shifts, and extrinsic calibration parameters.

Focused adjustments improve SLAM effectiveness for particular operational needs. KFS-LIO [138] applies a Dilution of Precision (DOP)-inspired criterion to quantitatively evaluate the geometric impact of each LiDAR feature constraint, removing the least informative constraints and significantly reducing computational demands during optimization. LIO-Vehicle [139] integrates non-holonomic motion constraints derived from the kinematics of ground vehicles, leading to a notable enhancement in trajectory accuracy for wheeled platforms operating on structured terrain.

Various systems combine advanced semantic, appearance-driven, and learned signals within factor graph optimization. Semantic-LIO [140] uses semantic segmentation to eliminate dynamic objects by removing points belonging to moving objects before feature extraction and optimization, reducing misleading constraints from dynamic scene elements. An innovative approach is introduced in [23] that enhances SLAM by utilizing intensity-augmented techniques for solid-state LiDARs. This method focuses on extracting both geometric and intensity edge points, employing multi-weighting functions that simultaneously account for geometry and intensity through iterative re-weighted least-squares optimization.

RF-LIO [141] adopts a removal-first strategy for highly dynamic environments. It uses a rough pose estimate from the LiDAR-inertial odometry pipeline together with adaptive multi-resolution range images to remove moving objects before scan-to-map matching, improving pose estimation when dynamic objects dominate the scene. S-Graphs+ [142] presents a hierarchical factor graph composed of four interconnected layers: robot poses, wall planes, room layouts, and floor levels. This framework incorporates semantic priors to produce structurally coherent indoor maps that cover multiple floors. LIO-SEGMOT [143] enhances LIO-SAM by integrating object detections through 3D multi-object tracking (MOT) constraints, utilizing an asynchronous graph formulation that facilitates ongoing updates of object states throughout robot keyframes.

In addition to strictly tightly coupled LiDAR-inertial graph-based estimators, several related graph-based or back-end SLAM frameworks are relevant because they address global consistency, scalability, map deformation, and multi-robot operation. Although these methods do not all formulate LiDAR and IMU measurements as tightly coupled factors within the same estimator, they provide important design patterns for graph optimization and large-scale SLAM. BALM [144] formulates local LiDAR bundle adjustment over a sliding window by minimizing distances from feature points to matched planar or edge structures. It analytically eliminates the feature parameters so that numerical optimization depends only on the scan poses, reducing the problem size and enabling real-time map refinement. Therefore, BALM serves as a back-end optimization strategy complementing the LiDAR-inertial coupled framework.

RDC-SLAM [145], ColaSLAM [146], and LAMP 2.0 [147] extend graph-based SLAM toward distributed, collaborative, or multi-robot operation, emphasizing communication efficiency, map fusion, and robust loop-closure management. Map-centric SLAM [148] departs from conventional pose-graph formulations by maintaining a global surfel map and applying elastic map deformation for loop-closure correction. LoLa-SLAM [149] targets low-latency LiDAR SLAM through continuous scan slicing and parallel registration, providing a latency-oriented LiDAR-only formulation rather than a tightly coupled LiDAR-inertial graph optimizer. These systems are therefore best understood as complementary graph-based or scalable SLAM extensions that inform the broader design space around tightly coupled LiDAR-inertial SLAM.

Recent contributions also explore specialized graph-based formulations for specific sensing conditions, map structures, and deployment constraints. LIPS [150] models environments using infinite planes and employs a singularity-free Closest Point (CP) parameterization to establish plane-to-plane constraints. NV-LIO [151] uses normal-vector-based registration derived from depth-image representations, improving alignment in confined environments such as narrow corridors and multi-floor buildings. GLIM [152] integrates GPU-accelerated scan-matching factors with keyframe matching and fixed-lag smoothing for efficient range-inertial localization and mapping. FF-LINS [153] addresses solid-state LiDARs with non-repetitive scan patterns through frame-to-frame registration and online calibration of LiDAR-IMU extrinsic and temporal offsets, avoiding reliance on explicit geometric feature extraction. LIMOT [154] advances dynamic-scene LiDAR-inertial estimation by combining LiDAR-inertial odometry with multi-object tracking. It uses IMU preintegration, CenterPoint-based object detection, trajectory-guided dynamic point filtering, and sliding-window factor graph optimization to jointly estimate ego-motion and moving-object trajectories.

In contrast to filter-based frameworks, graph-based tightly coupled systems provide enhanced global consistency and greater flexibility in sensor data fusion by utilizing delayed optimization and loop closure techniques. Although they require more computational resources, these methods provide enhanced accuracy, robustness, and extensibility, which are essential for extensive, dynamic, and prolonged robotic operations.

Table A6 in the Appendix summarizes representative graph-based tightly coupled systems. Across these methods, a consistent pattern emerges: systems with explicit loop closure or global optimization, such as LIO-SAM [13], LVI-SAM [130], CLINS [135], Wildcat SLAM [136], and DMSA-SLAM [127], target reduced drift in revisited environments, whereas local-window or odometry-oriented methods prioritize real-time consistency, specialized sensing constraints, or platform-specific robustness.

### 5.3. Taxonomic Synthesis of LiDAR-Inertial Architectures

Table 3 synthesizes the LiDAR-inertial methods discussed above according to how LiDAR and inertial measurements enter the estimator. It distinguishes LiDAR-dominant loosely coupled pipelines, tightly coupled recursive estimators, and tightly coupled graph or window-based optimization methods. The comparison focuses on state coupling, computational growth, retrospective correction, loop-closure support, and principal failure modes. Representative systems are summarized in Appendix Table A4, Table A5 and Table A6.

#### Comparative Analysis of LiDAR-Inertial Integration Paradigms

The transition from standalone LiDAR odometry to LiDAR-inertial fusion helps address geometric degeneracy and intra-scan motion distortion [10]. Loosely coupled systems such as LeGO-LOAM [84] and Adaptive-LIO [86] maintain modular LiDAR-dominant pipelines in which IMU measurements assist initialization, de-skewing, ground processing, or fault-aware mode switching without being jointly optimized with LiDAR residuals. As detailed in Section 5.1, this preserves modularity but prevents LiDAR updates from directly refining IMU biases. Runtime and drift should therefore be evaluated per implementation rather than inferred solely from the coupling category.

Figure 8a,b present the comparisons reported in the DLIO study for Newer College Short and UCLA Campus A datasets, respectively, under a common reporting source and hardware platform [115]. Since the methods differ in estimator design, registration, mapping, and configuration, the results provide a source-conditioned performance comparison rather than a controlled isolation of inertial integration or continuous-time motion correction. On the Newer College Short sequence, LiDAR-only DLO and CT-ICP report absolute trajectory error (ATE) RMSE values of 0.4633 and 0.5552 m, respectively, while LIO-SAM, FAST-LIO2, and DLIO report 0.3957, 0.3775, and 0.3606 m [115]. On the UCLA Campus A sequence, DLIO reports the lowest end-to-end translational error at 0.0105 m, compared with 0.0216 m for DLO and LIO-SAM, 0.0387 m for CT-ICP, and 0.0454 m for FAST-LIO2 [115]. Because the two sequences use different error metrics, values are compared only among methods within each sequence panel in Figure 8a,b separately.

Beyond accuracy, the estimator structure within tightly coupled LIO influences update latency and retrospective correction capability. The FAST-LIO2 architecture described in Section 5.2.1 reports approximately 1.82 ms per scan on an Intel platform, 5.23 ms on ARM, and 19.6–23.9 ms in airborne experiments [88], demonstrating the dependence of reported latency on platform and experiment. LINS [105], LIO-EKF [109], and SR-LIO [110] address different constraints, including local-frame error-state estimation, high-frequency filtering, and sweep-distortion handling, while retaining recursive state updates.

In purely recursive filtering formulations, past states are normally marginalized or removed from the active state and are not directly reoptimized using later measurements. This limits retrospective correction within the filter itself, although a separate pose-graph or smoothing back-end may still revise the global trajectory. Optimization-based systems retain selected historical states so that delayed constraints can revise earlier estimates. LIO-SAM [13] uses iSAM2 incremental smoothing instead of batch nonlinear optimization, and accepts IMU-preintegration, LiDAR-odometry, GPS, and loop-closure factors. Acting as a back-end optimization method, BALM [144] applies bundle adjustment to plane-feature constraints rather than jointly optimizing LiDAR and inertial states. Its reported local 20-scan bundle optimization operates near 10 Hz, illustrating a back-end optimization strategy but not a tightly coupled LIO architecture. CLINS [135] represents continuous-time LiDAR-inertial optimization with staged loop correction. These systems therefore represent incremental smoothing, LiDAR bundle adjustment, and continuous-time estimation rather than one uniform batch-graph category.

Map structure further differentiates recursive estimators. FAST-LIO2’s ikd-Tree supports insertion, deletion, on-tree downsampling, and dynamic rebalancing, providing accurate local association at the cost of continued tree maintenance, lazy deletion, and rebalancing [88]. Faster-LIO’s iVox replaces exact global k-nearest-neighbor search with hash-indexed nearby-voxel search. It reports processing frequencies of 1000–2000 Hz for solid-state LiDAR and more than 200 Hz for 32-line spinning LiDAR on CPU, together with almost 40% processing-time savings on NCLT and UTBM [89]. Its approximate search remains dependent on voxel size, point density, and neighboring-voxel selection. LIO-GVM [102] stores centroids and covariances in hashed Gaussian voxels for covariance-based residuals, whereas iG-LIO [98] estimates surface covariances from neighboring voxels to avoid repeated kd-tree range searches. At the global level, LIO-SAM [13] controls graph and map growth through selective keyframes and fixed-size sub-keyframe maps. Sparse keyframes reduce memory and computation but may lose geometric coverage, whereas dense keyframes improve coverage at the cost of larger maps and heavier optimization.

The practical outcomes of these architectural and processing choices can be examined through source-conditioned benchmark comparisons. Figure 8c summarizes the comparison reported in SR-LIO Table III for the first five NCLT sequences [110]. The SR-LIO authors generated all comparison results from author-provided source code and ran the experiments on the same Intel Core i7-12700/32 GB platform, permitting a within-study comparison over a common sequence set. Mean ATE RMSE is 1.81 m for SR-LIO, 1.98 m for iG-LIO, 2.88 m for FAST-LIO2, 4.66 m for Point-LIO, and 6.33 m for DLIO. LIO-SAM obtains 3.47 m over four completed sequences but fails on the 2012-05-11 sequence. Its RMSE is only representative of four out of five sequences compared to the other approaches. DLIO’s mean is driven mainly by its 18.10 m error on the fourth sequence, reinforcing the need to report per-sequence errors and failures alongside aggregate means.

Finally, Figure 8d summarizes the average per-frame processing times reported by Adaptive-LIO for the three Robot Town sequences [86]. Across the three sequences, the reported means are 4.9 ms for iG-LIO, 5.9 ms for DLIO, 9.9 ms for Point-LIO, 10.4 ms for FAST-LIO2, 31.2 ms for Adaptive-LIO, and 57.5 ms for LIO-SAM. The 50 ms line denotes the nominal per-frame budget for 20 Hz operation. These values must be interpreted jointly with accuracy and sequence completion: LIO-SAM failed all three sequences, while iG-LIO failed on sequence C. Consequently, timing reported for an unsuccessful run does not establish successful real-time localization. These outcomes demonstrate that low processing time does not by itself establish robust real-time operation and that estimator category alone does not determine latency. Implementation design, map representation, optimization scope, experimental configuration, and sequence completion must therefore be considered together. The Robot Town results consequently show an implementation-specific trade-off among estimator structure, runtime, and successful sequence completion rather than a universal computational advantage of filtering over graph optimization.

Selected results underlying Figure 8 are compiled in Appendix Table A7, together with the corresponding architecture, metric, hardware, and protocol conditions, and completion status. Taken together, coupling strategy, estimator structure, and map representation jointly influence accuracy, latency, and correction capability. None of these architectural dimensions alone predicts overall system performance.

## 6. Global SLAM Functions and Benchmarking Context

The previous sections classified LiDAR-only and LiDAR-inertial SLAM systems by sensing modality, architectural coupling, and localized state estimation trade-offs. This section shifts from local odometry to the sensor-agnostic components that support long-term autonomous deployment, such as global consistency, prior-map localization, and scalable mapping, followed by benchmark datasets and application scenarios.

### 6.1. Place Recognition Descriptors and Loop Closure

Loop closure detection (LCD) mitigates long-term odometry drift by recognizing previously visited locations and adding non-sequential constraints to the back-end optimizer. While scan matching provides local consistency, small errors accumulate over long trajectories. LCD determines whether a SLAM system can maintain global map consistency during extended operation. Most LCD pipelines include two stages: place recognition, which retrieves candidate revisits from scans, keyframes, submaps, or descriptors, and geometric verification, which validates the candidate and estimates a relative transformation for pose-graph correction. This separation is critical because high retrieval recall alone is insufficient: false loop closures can corrupt the map, while overly conservative verification allows drift to persist.

LCD methods have progressed from handcrafted geometric descriptors toward learned, multimodal, and distributed formulations. Scan Context [25] remains influential because it encodes a LiDAR scan as a polar descriptor with efficient ring-key retrieval and yaw alignment, providing a practical balance between efficiency and geometric interpretability. Learned descriptors replace handcrafted geometry with data-driven global representations. OverlapTransformer [155] uses range-image projection, transformer-based feature aggregation, and NetVLAD to produce yaw angle-invariant descriptors, reporting a 256-dimensional descriptor and approximately 1.37 ms extraction time per scan, or about 730 Hz, on an Intel i7-11700K and NVIDIA RTX 3070 setup. BEVPlace [156] and BEVPlace++ [157] use bird’s-eye view representations. BEVPlace reports 99.3% mean Recall on KITTI and approximately 30 ms per query on an RTX 3090 GPU, while BEVPlace++ adds rotation-equivariant local features and RANSAC-based 3-DoF pose estimation, reporting a 17 MB model, more than 20 Hz desktop global localization, and approximately 10 Hz operation on an NVIDIA AGX ORIN. These methods show that learned LCD is moving from offline retrieval toward deployable loop-closure integration, although performance remains tied to training domain, sensor configuration, and hardware.

Multimodal and verification-focused methods address different weaknesses in the LCD pipeline. UMF [158] fuses visual and LiDAR features through cross-attention and re-ranking, improving retrieval in aliased or weakly structured environments, with reported Recall of 75.3% on DLR S3LI and 98.3% on Oxford RobotCar using its RANSAC re-ranking variant. However, multimodal re-ranking increases computational cost, calibration dependence, and system complexity. At the verification stage, RANSAC remains widely used because it is simple and effective when enough correct correspondences are available [159], but it can fail under sparse correspondences, low inlier ratios, or perceptual aliasing [160]. CliReg [160] addresses this by replacing RANSAC with deterministic maximal-clique search over a compatibility graph. On challenging HeLiPR sequences, it recovers consistent inlier sets in cases where RANSAC-10K fails, and reports 2.8–6.3 ms per match in 3D experiments. This improves verification reliability but introduces graph-search complexity because maximal clique search is NP-hard.

In long-term and multi-robot mapping, loop closure also becomes a communication and robust-optimization problem. Wildcat SLAM [136] uses submap-based pose-graph optimization for large-scale and multi-agent mapping. RDC-SLAM [145] emphasizes distributed cooperative 3D LiDAR SLAM under limited communication. ColaSLAM [146] moves part of collaborative mapping to edge servers. LAMP 2.0 [147] combines scalable loop-closure detection with robust back-end optimization using Graduated Non-Convexity for subterranean multi-robot SLAM. These systems show that loop closure at scale requires not only reliable place recognition and verification, but also efficient data exchange, scheduling, and outlier handling.

Overall, LCD methods differ in the stage of the pipeline they strengthen. Scan Context improves efficient geometric retrieval. OverlapTransformer, BEVPlace, and BEVPlace++ improve learned place recognition and relative-pose estimation. UMF improves robustness through multimodal fusion, while CliReg strengthens geometric verification, and multi-robot systems extend LCD to distributed mapping. The central trade-off is among recall, precision, and verification costs. High recall is needed to correct drift, while high precision is needed to avoid corrupting the pose graph, and robust verification or multimodal fusion often increases computation, training requirements, calibration sensitivity, or communication overhead. Robust LCD therefore remains one of the main distinctions between short-term odometry systems and SLAM systems capable of long-duration, multi-session, or collaborative mapping.

### 6.2. Prior-Map Localization and Relocalization

Global localization estimates an initial pose in a previously constructed map without requiring an accurate pose prior. Prior-map localization tracks the platform relative to that map. Relocalization re-establishes the global pose after local tracking becomes unreliable. These methods differ from loop closure, which detects a revisit within the active trajectory to constrain accumulated drift. Nevertheless, loop closure and relocalization commonly employ the same coarse-to-fine sequence, in which candidate retrieval is followed by geometric verification and relative pose estimation. When incorporated into a SLAM system, the recovered pose may reinitialize the local estimator, introduce a global constraint into a pose graph, or align the active submap with a persistent map. An incorrect association can instead produce pose discontinuity or corrupt the global estimate.

Existing methods and systems implement these functions through several architectural mechanisms. LOL [44] combines LiDAR odometry with localization in a prebuilt point cloud map, whereas LocNDF [78] performs scan registration and Monte Carlo localization against a neural distance-field map. EgoNN [161] explicitly separates global descriptor candidate retrieval from local feature matching and robust 6-DoF pose estimation. Similarly, LCR-Net [162] combines retrieval and registration but additionally integrates tracking-degeneration detection and relocalization into a complete LiDAR SLAM pipeline. Within LiDAR-inertial estimation, the SRIF-based method [111] performs localization against a prebuilt feature map, while Koide et al. [163] tightly couple scan-to-scan, scan-to-map, and IMU factors in a sliding window graph. The latter maintains local tracking through defective or unmapped map regions and reactivates map constraints when overlap becomes available, although it does not implement general robot recovery. LIO-Fusion [131] instead introduces validated GNSS or relocalization measurements as global factors within a LiDAR-inertial factor graph. These approaches demonstrate that prior-map localization and relocalization can be incorporated into LiDAR-only and LiDAR-inertial pipelines through retrieval and registration modules, learned map representations, recursive estimators, or factor graphs without constituting a separate sensing architecture.

Long-term operation introduces an additional problem: the prior map may become incomplete or inconsistent with the current environment. Ding et al. [164] combine global map matching with LiDAR-inertial odometry in a pose graph framework and detect map regions affected by environmental change. LT-mapper [165] addresses multi-session alignment, change detection, and persistent map management over temporal intervals ranging from days to years. LTA-OM [166] uses long-term associations and prior-map factors for multi-session relocalization and map alignment, whereas SLAM-RAMU [167] combines initial alignment, repeated localization, and selective map updating. Accordingly, prior-map localization, relocalization, and map updating are best treated as efficient global alignment and recovery approaches rather than replacements for the LiDAR-only and LiDAR-inertial taxonomy.

Evaluation should distinguish the stages at which failure can occur. Candidate retrieval should report recall and false-positive behavior. Pose recovery should report success thresholds, translational and rotational accuracy, and unsuccessful registrations, whereas system-level evaluation should report recovery latency, trajectory continuity after reinsertion, and subsequent tracking stability. Long-term evaluations should additionally examine operation with stale, partially changed, incomplete, or temporarily unavailable map regions. Failed cases must remain visible because accuracy computed only over successful re-localizations does not describe end-to-end recovery reliability.

### 6.3. Map Scalability and Optimization Back-Ends

As systems transition from local tracking to global SLAM, map design and solver architecture become major factors affecting long-term memory growth and optimization latency. Adaptive map resolution is important because fixed voxel parameters often fail across indoor, outdoor, and transitional environments. Adaptive-LIO [86] introduces a distance-dependent multi-resolution voxel map using representative resolutions of 0.2, 0.5, and 1.2 m to preserve nearby detail while representing more distant measurements more coarsely. This improves mapping quality in narrow rooms and hallways and during transitions between environments, but increases computation, with reported processing times of approximately 25–35 ms per frame in the relevant experiments. GenZ-LIO [97] extends this idea through scale-aware adaptive voxelization, using scene scale and a PD controller with sensitivity-informed gain scheduling to adjust voxel size. This helps avoid divergence in confined spaces where fixed voxelization can leave too few usable points.

R-VoxelMap [96] and VoxelMap++ [95] further show that voxel-map scalability depends not only on global voxel size but also on internal voxel structure and inter-voxel relationships: R-VoxelMap uses recursive octree subdivision and RANSAC-based plane fitting to preserve mixed structures, while VoxelMap++ merges coplanar voxel planes using union-find to reduce fragmentation and redundant storage in large planar environments. Related voxel representations also reduce the cost of local estimation. iG-LIO [98] uses a voxel-based surface covariance estimator and an incremental voxel map to reduce covariance computation, nearest-neighbor search, and map-management costs. LIO-GVM [102] instead represents each voxel using a centroid and covariance matrix and incorporates Gaussian-statistics-based residuals into registration. These systems demonstrate that voxel structure affects not only map storage but also the efficiency and robustness of online data association.

Implicit neural representations provide a more recent alternative for compact and continuous mapping. SHINE-Mapping [77] is a method that stores sparse hierarchical implicit features in an octree-based structure and decodes signed-distance values using a shallow neural network, reporting an example memory footprint of 84 MB. Although it assumes provided poses, its compact, continuous representation illustrates how an implicit mapping module could reduce map-storage requirements and support detailed surface reconstruction within a broader SLAM system. PIN-SLAM [21] demonstrates the scalability benefits of an elastic implicit representation within a complete SLAM system. Its sparse neural-point map can deform with global pose adjustments, allowing loop-closure corrections to be reflected in the reconstructed map while retaining compact storage. In one reported example, the PIN map occupied 0.5 GB compared with 183 GB for the corresponding raw point cloud. KN-LIO [122] further couples neural fields with LiDAR-inertial estimation using point-SDF residuals in semi-coupled and tightly coupled formulations. These methods improve compactness and surface continuity, but shift cost toward online neural-field optimization, SDF querying, and GPU-dependent deployment. Taken together, map representation is a central source of the trade-off among accuracy, scalability, memory, and real-time operation.

Beyond map representation, back-end optimization design introduces another source of latency and scalability constraints. The reviewed systems show that formulation and constraint construction often matter more than the solver library alone. LIO-SAM uses iSAM2 through GTSAM [13], CT-ICP and ART-SLAM use g2o for pose-graph optimization [22,60], and CLINS and T-LOAM use Ceres for nonlinear least squares [46,135]. Their computational behavior therefore cannot be attributed to the solver libraries alone, because residual design, state dimension, robust losses, optimization scope, and global-correction strategy also determine latency and scalability.

### 6.4. Benchmark Datasets and Application Scenarios

Benchmark selection determines which conclusions a SLAM evaluation can support because datasets differ in platform motion, sensor configuration, ground-truth quality, environmental change, and represented failure mechanisms. Table 4 groups representative datasets and evaluation evidence according to their application scenario, dominant challenge, typical validation purpose, and remaining validation gap.

It is important to mention that 4Seasons [172] does not provide onboard LiDAR measurements in its dataset; instead, it provides stereo images, IMU measurements, and RTK-GNSS, with globally consistent reference poses generated by combining stereo visual-inertial odometry with RTK-GNSS. Similarly, EuRoC [175] provides synchronized stereo-inertial measurements rather than onboard LiDAR, together with Vicon 6-DoF poses, Leica laser tracker positions, and Leica 3D structure scans. Nevertheless, these datasets provide accurate trajectory and structural references, as well as challenging operating conditions, supporting ground-truth comparison, trajectory assessment, map alignment, and evaluation protocol design.

## 7. Discussion: Open Challenges and Research Directions

The analysis in Section 4, Section 5 and Section 6 shows that trajectory accuracy on individual benchmark sequences is insufficient to establish deployment readiness. LiDAR-only and LiDAR-inertial systems may achieve low average error while remaining vulnerable to silent estimator inconsistency, environmental transitions, stale map content, or cumulative resource growth. These limitations concern five interrelated problem domains: the internal state of the estimator, the operating conditions encountered at runtime, the evolution of the represented world across sessions, the resources available to sustain operation, and the interface through which localization and mapping information supports downstream navigation and motion planning.

### 7.1. Trustworthy Estimation and Autonomous Recovery

Calibration approaches range from dedicated targetless procedures and automated startup initialization to joint estimation during odometry. Observability-aware LiDAR-IMU calibration [180], GRIL-Calib [181], and the LM-Calibr component in [120] estimate sensor or actuator parameters from motion data collected during dedicated calibration procedures. LI-Init [182] instead embeds a finite, excitation-aware initialization phase into system startup and supplies the estimated LiDAR-IMU extrinsics, temporal offset, gravity direction, and IMU biases to the subsequent LIO estimator. LIC-Fusion 2.0 [183] and OMC-SLIO [121] go further by retaining calibration variables within the operating estimator and refining them jointly with odometry. These online approaches reduce dependence on accurately known pre-calibration but do not eliminate the need for adequate initialization, informative motion, and suitable environmental constraints.

Despite this progression, parameter estimation alone does not provide long-term calibration health management. Apparent calibration changes may reflect physical sensor displacement or thermal and mechanical effects, but they may also arise from insufficient excitation, trajectory or timing errors, and incorrect data association. Future systems must distinguish genuine calibration change from these estimation artifacts before deciding whether calibration states should be updated, frozen, or reinitialized. This requires observability-aware update gating, propagation of calibration uncertainty into residual weighting and sensor-fusion decisions, and independent verification that recalibration improved estimator consistency. Failed updates should trigger rollback or an appropriate fallback rather than being incorporated into the operating state.

Uncertainty representation remains similarly fragmented. LOG-LIO2 [100], LIO-GVM [102], and LIO-EKF [109] model uncertainty in selected odometry, map, or data-association components, while factor-graph formulations encode assumed measurement uncertainty and continuous-time formulations represent motion between measurement timestamps [26,135,136]. However, pose covariance alone does not capture uncertainty in calibration, IMU propagation, LiDAR residuals, correspondences, map elements, loop closures, and global corrections. Future research should connect these sources across the estimation pipeline so that uncertainty can inform failure prediction, measurement selection, map updates, and downstream navigation. Operationally, confidence bounds are calibrated when they achieve their stated empirical coverage, while predicted risk should both correspond to observed failure frequencies and distinguish degraded from nominal operation.

Existing systems provide partial failure awareness. Adaptive-LIO [86], DAMS-LIO [119], and Switch-SLAM [184] detect degraded conditions and respectively adapt motion estimation, incorporate auxiliary odometry, or switch between LiDAR and visual odometry, while DALI-SLAM [185] combines degeneracy-aware estimation with distortion correction and pose-graph constraints. Detection or mode switching alone, however, does not demonstrate autonomous recovery. A complete reliability process must detect degradation, diagnose its probable cause, limit the influence of unreliable measurements, select an appropriate fallback, recover a consistent estimate, and verify that normal operation can safely resume. Major unresolved risks include false alarms, incorrect fault attribution, delayed response, unstable switching, and premature termination of recovery.

Accordingly, progress should be judged by whether a system can detect, contain, and recover from controlled calibration, sensing, association, and degeneracy failures, rather than by its final trajectory error alone, as indicated in Table 5.

### 7.2. Generalization Across Environments and Sensor Conditions

Section 4 and Section 5 show that feature extraction, correspondence selection, voxel resolution, robust losses, keyframe policies, and modality weights are commonly fixed or tuned for particular environments. Such configurations can produce strong results on homogeneous sequences but degrade at environmental changes such as indoor–outdoor transitions, corridors and tunnels, forests, open areas, repetitive structures, dynamic urban scenes, or adverse sensing conditions. This problem differs from the internal failure diagnosis considered in Section 7.1. Environmental-change detection identifies a change in operating conditions. Failure diagnosis determines whether that change has already made the estimator unreliable. A generalizable system must perform both tasks without confusing a legitimate environmental transition with a sensor fault.

Adaptive-LIO [86] demonstrates observability-aware adaptation, while GenZ-LIO [97] targets generalization across confined and open environments through scale-adaptive voxelization. These studies show that runtime adaptation can use observability, IMU condition, scene scale, and point density. Other candidate signals include residual distributions, constraint rank, correspondence consistency, motion characteristics, and sensor reliability. The unresolved question is how to convert those signals into stable changes in feature selection, association thresholds, robust losses, map resolution, keyframe insertion, or modality contribution. Otherwise, adaptation can oscillate near decision boundaries, react too slowly to abrupt transitions, or overfit to temporary conditions.

Semantic, motion-aware, learned, and structural components are potential inputs to these decisions, rather than future objectives in themselves. RF-LIO [141], Semantic-LIO [140], and LIMOT [154] show that object and motion information can support dynamic-object filtering and tracking, while S-Graphs+ [142] shows that structural information can support localization and mapping. Yet semantic class alone is insufficient. A currently stopped vehicle may provide useful short-term geometry, while a nominally static structure may be undergoing construction. Semantic confidence and temporal object state should therefore influence, but not deterministically control, measurement weighting and map updates. Learned components can also introduce dataset bias, domain shift, platform dependence, and uncertain behavior outside their training distributions. Unsafe online adaptation may reinforce incorrect correspondences or semantic predictions, producing a self-confirming failure.

As indicated in Table 5, evaluation should therefore determine whether adaptation remains timely and stable during within-sequence transitions and generalizes across datasets and platforms relative to fixed-parameter baselines.

### 7.3. Persistent World Models and Lifelong Map Maintenance

Runtime adaptation does not address the separate problem that the world model itself becomes outdated. A single-session map may contain parked vehicles, vegetation, temporary barriers, construction activity, or structures that later appear, disappear, or change state. Removing currently moving objects is insufficient because many relevant changes occur between sessions rather than during a single traversal. The central problem is therefore how to preserve a reliable world model while distinguishing real environmental change from localization, registration, association, or estimation error.

Recent systems address complementary requirements of persistent world-model maintenance. LT-mapper [165] combines multi-session SLAM with dynamic-change detection and positive and negative change management. LTA-OM [166] integrates long-term association mapping with multi-session map reuse and relocalization, whereas SLAM-RAMU [167] combines prior-map relocalization with selective map updating based on historical observations. CAOM [186] aligns current LiDAR observations with a high-precision prior map and probabilistically fuses detected changes across observations. ELite [187] uses ephemerality modeling to align sessions, remove transient elements, and maintain an updated map, while Chamelion [188] performs occlusion-aware online change detection and confidence-weighted prior-map updating. Together, these systems demonstrate progress from static single-session maps toward persistent maps that can be aligned, reused, revised, and maintained across sessions. However, integrating reliable change interpretation, uncertainty-aware updating, historical retention, and controlled forgetting within a unified and broadly validated world model remains an open problem.

A persistent world model must represent not only geometric changes but also their confidence, temporal persistence, and provenance. Map updates should be gated by localization confidence and repeated observations, with uncertain changes retained provisionally rather than immediately overwriting prior content. Versioning, rollback, and bounded-retention policies are also needed to recover from incorrect updates, limit storage growth, and preserve information required for long-term relocalization.

Semantic representations may help identify transient objects, while neural representations may provide compact, continuous maps, as illustrated by PIN-SLAM [21]. However, their suitability for lifelong operation should be evaluated against explicit geometric baselines, considering update cost, storage growth, temporal consistency, relocalization accuracy, uncertainty, catastrophic forgetting, and preservation of stable structure.

Therefore, lifelong mapping should be assessed by separating localization error from change-classification error and by measuring relocalization accuracy, map-update rate, stale-map failures, and storage growth across repeated sessions, as summarized in Table 5.

### 7.4. Resource-Bounded and Collaborative Operation

Section 4, Section 5 and Section 6 identify several methods that reduce per-frame cost, including incremental spatial indexing in FAST-LIO2 [88], sparse voxel structures in Faster-LIO [89], mergeable voxel maps in VoxelMap++ [95], and compact implicit representations in PIN-SLAM [21]. These results are important, but per-frame efficiency does not establish long-term scalability. Computation may grow with the local optimization window, map memory with mission duration and mapped area, relocalization cost with the number of stored places, and global correction cost with the number of keyframes or submaps. Neural or implicit representations may compress geometry but still incur online optimization cost, representation drift, or dependence on high-end GPUs.

The central research question is how to preserve localization accuracy, map utility, relocalization capability, and global consistency under fixed computation, memory, energy, and communication budgets. Candidate directions include bounded-memory local maps, uncertainty-aware marginalization, selective retention of critical relocalization regions, hierarchical submaps, asynchronous or selective global optimization, and compression that preserves both geometry and place-recognition information. Resource policies should be connected to estimator uncertainty and mission objectives: removing the oldest information is not necessarily safe if that information provides the only reliable constraint for later relocalization. Tail latency is also important because occasional optimization spikes can violate real-time deadlines even when average runtime remains acceptable.

Collaborative systems introduce additional scaling dimensions. Wildcat [136], RDC-SLAM [145], ColaSLAM [146], and LAMP 2.0 [147] demonstrate multi-session or multi-robot estimation, exchange of mapping information, distributed or edge-assisted processing, and robust global alignment. Remaining challenges include determining which submaps, descriptors, or constraints should be transmitted. Systems must also maintain consistency despite delayed or missing inter-robot measurements, reject erroneous inter-robot loop closures, and accommodate differences in sensors, computational resources, and map representations. Adding more agents improves mapping only when the additional observations justify the associated communication and global consistency costs.

Therefore, scalability claims should report, with complete hardware and sensor disclosure, how runtime, memory, and communication grow with mission duration, mapped area, and the number of sessions and agents, rather than relying on average per-frame runtime alone, as indicated in Table 5.

### 7.5. Planning Interface for Autonomous Navigation

In autonomous navigation, SLAM and motion planning are treated as interacting rather than strictly sequential modules. The SLAM module can be responsible for providing the estimated pose and environmental map, together with any available information concerning uncertainty, map validity, and estimator status. The executed motion changes the sensor viewpoint, vehicle excitation, and future observability of the environment. Active SLAM formalizes this relationship by selecting actions that improve localization or mapping information [189]. Therefore, a planner that treats the current pose and map as exact may select a geometrically feasible route through regions in which localization is poorly constrained. For example, Valencia et al. [190] provide an early example by using a Pose SLAM graph as a belief roadmap and incorporating accumulated pose uncertainty into route selection.

Existing studies illustrate complementary mechanisms for strengthening this interface. Motion and uncertainty-aware planning can select dynamically feasible trajectories that preserve estimator observability and avoid motions under which the vehicle’s state becomes poorly constrained [191]. SLAP instead performs dynamic replanning in belief space as the robot belief, obstacle map, or goal changes, including after recovery from large pose deviations [192]. Perception-aware planning provides another mechanism by steering the platform away from regions expected to degrade state estimation and maintaining informative landmarks within the sensor field of view [193]. These studies are not all LiDAR-specific and are included as architectural examples of the localization-planning interface. For LiDAR and LIO systems, corresponding interface information may include calibrated pose confidence, geometric-degeneracy indicators, registration quality, local-map completeness, map age, and relocalization or recovery status.

Motion planning must also satisfy vehicle and environmental constraints independently of estimator quality. Curvature-constrained Dubins planning for forward-only vehicles provides an example of this requirement. The unified approach in [194] provides a quality-based treatment of obstacle avoidance under curvature constraints. The review in [195] summarizes developments in Dubins planning involving obstacles, variable speed, cooperative operation, and real-time execution. The relevance of these to SLAM is that a reliable pose and map remain insufficient unless they support collision-free and dynamically feasible motion. Therefore, an integrated LiDAR/LIO navigation system should expose estimator confidence and map validity to the planner, trigger replanning or safe fallback after degradation or global correction, and account for how the selected motion will affect subsequent estimation. Evaluation should jointly report localization and map error, task completion and collision outcomes, path length or execution time, curvature or dynamic feasibility, uncertainty along the executed path, and replanning and recovery latency.

### 7.6. Evidence Requirements and Research Roadmap

Absolute and relative trajectory error remain necessary evaluation metrics, but they summarize the final pose discrepancy rather than the mechanism by which a system remained reliable or failed. Low average error does not show that uncertainty was calibrated, a transition was detected promptly, a failed estimator recovered safely, a stale map was revised correctly, or resource use remained bounded. Evaluation must combine trajectory and map accuracy with measurements of failure and recovery, adaptation, long term map maintenance, resource use, and downstream navigation. Failed and incomplete sequences must be included rather than removed from aggregate results, and initialization behavior, hardware, sensor rates, sensor resolutions, and parameter changes should be disclosed. Table 5 synthesizes the five research directions developed above by linking each limitation identified in the review to its unresolved technical problem and the minimum evidence required to demonstrate progress. The ordering reflects the organization of the discussion rather than a ranking of scientific importance.

The five directions are interdependent but should not be collapsed into a single notion of robustness. Runtime adaptation requires trustworthy state assessment, while lifelong map revision requires reliable localization and calibrated update confidence, and both become operationally meaningful only when their resource requirements remain bounded. Downstream planning additionally depends on explicit information about pose and map validity, while the selected motion can influence subsequent localization and mapping quality. The resulting roadmap does not assume that one architecture, map representation, or learning paradigm will dominate LiDAR SLAM. Instead, it identifies five unresolved research problems and the minimum evidence required to demonstrate progress toward trustworthy, adaptive, persistent, resource-bounded, and navigation-ready systems.

## 8. Conclusions

This review examined LiDAR and LiDAR-inertial SLAM systems through a dual-axis, architecture-centered framework that distinguishes sensing configuration and architectural organization. Covering more than 100 representative systems published between 2014 and 2026, the review analyzed LiDAR-only methods, loosely coupled fusion, tightly coupled filter-based fusion, and tightly coupled graph-based fusion as well as supporting methods such as scan matching, loop closure, and map optimization.

The analysis shows that the field has progressed from modular LiDAR-based pipelines toward more tightly integrated estimation frameworks. Filter-based LiDAR-inertial methods provide low-latency state estimation and are well suited to real-time deployment, while graph-based and smoothing-based methods provide stronger mechanisms for loop closure, global consistency, and multi-factor optimization. Direct registration, continuous-time trajectory modeling, adaptive map representations, semantic reasoning, and neural implicit mapping have further expanded the design space. However, these gains are accompanied by increased algorithmic complexity, hardware demands, calibration sensitivity, and implementation burden.

A central finding of this review is that no single SLAM architecture satisfies all deployment requirements. Performance depends on the interaction between sensor configuration, motion profile, environmental structure, computational platform, map representation, and evaluation metric. Structured automotive benchmarks such as KITTI reveal convergence in trajectory accuracy among several LiDAR-only methods, whereas long-duration, adverse-weather, handheld, and industrial datasets expose stronger differences in robustness, runtime, and failure modes. Fair comparison requires aligned dataset subsets, metrics, evaluation protocols, and cross-dataset validation, with transparent reporting of the hardware specifications and runtime conditions.

The open challenges discussed in Section 7 indicate that future LiDAR-based SLAM research will increasingly depend on calibration-aware fusion, uncertainty propagation, failure detection and recovery, generalization across environments and sensor conditions, persistent map maintenance, resource-bounded operation, and dependable interfaces between localization, mapping, and downstream motion planning.

## Figures and Tables

**Figure 1:**
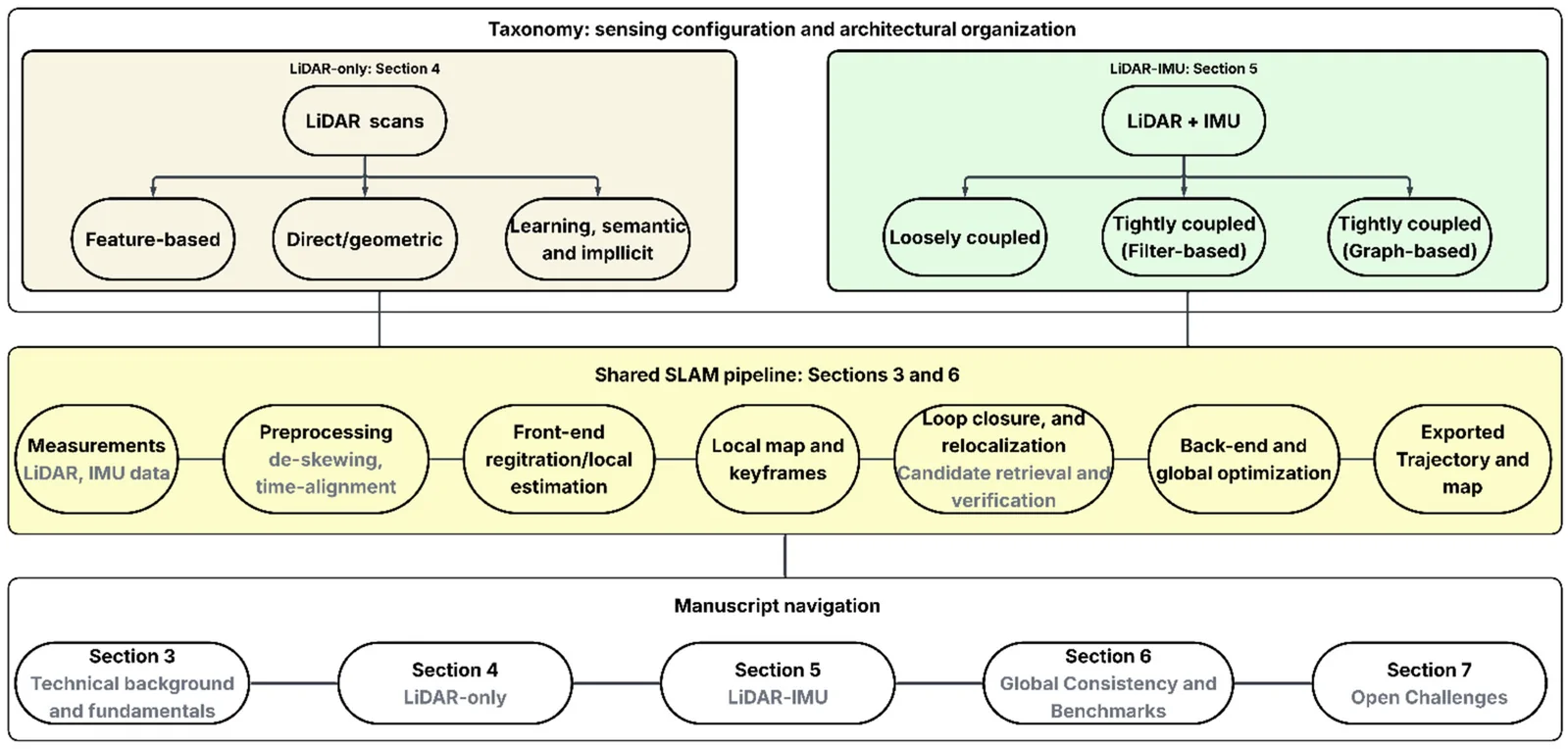
Architecture-centered taxonomy and organization of the review.

**Figure 2:**
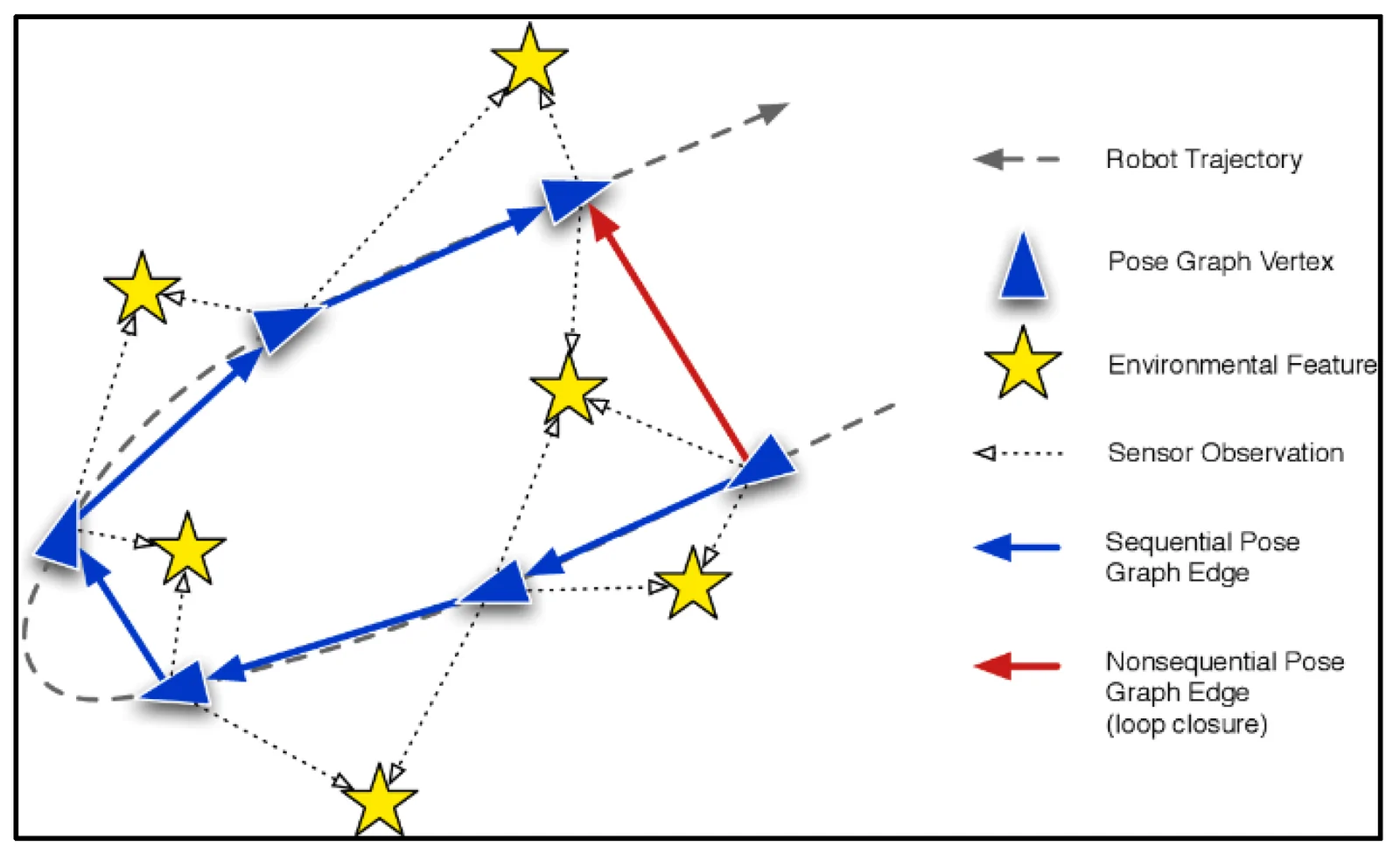
Illustration of the robot’s pose and trajectory [15].

**Figure 3:**
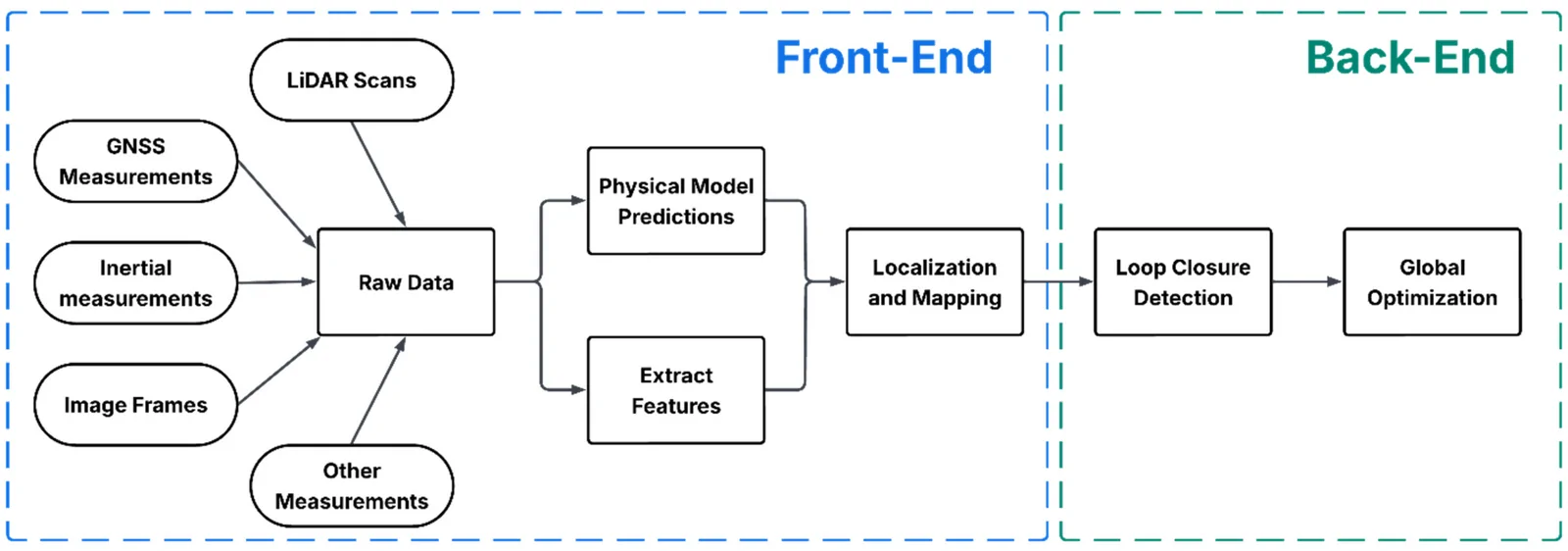
General multi-sensor front-end/back-end organization of a SLAM system. LiDAR, inertial, image, GNSS, and other measurements may contribute to the estimator depending on the sensing configuration (Redrawn from [9]).

**Figure 4:**
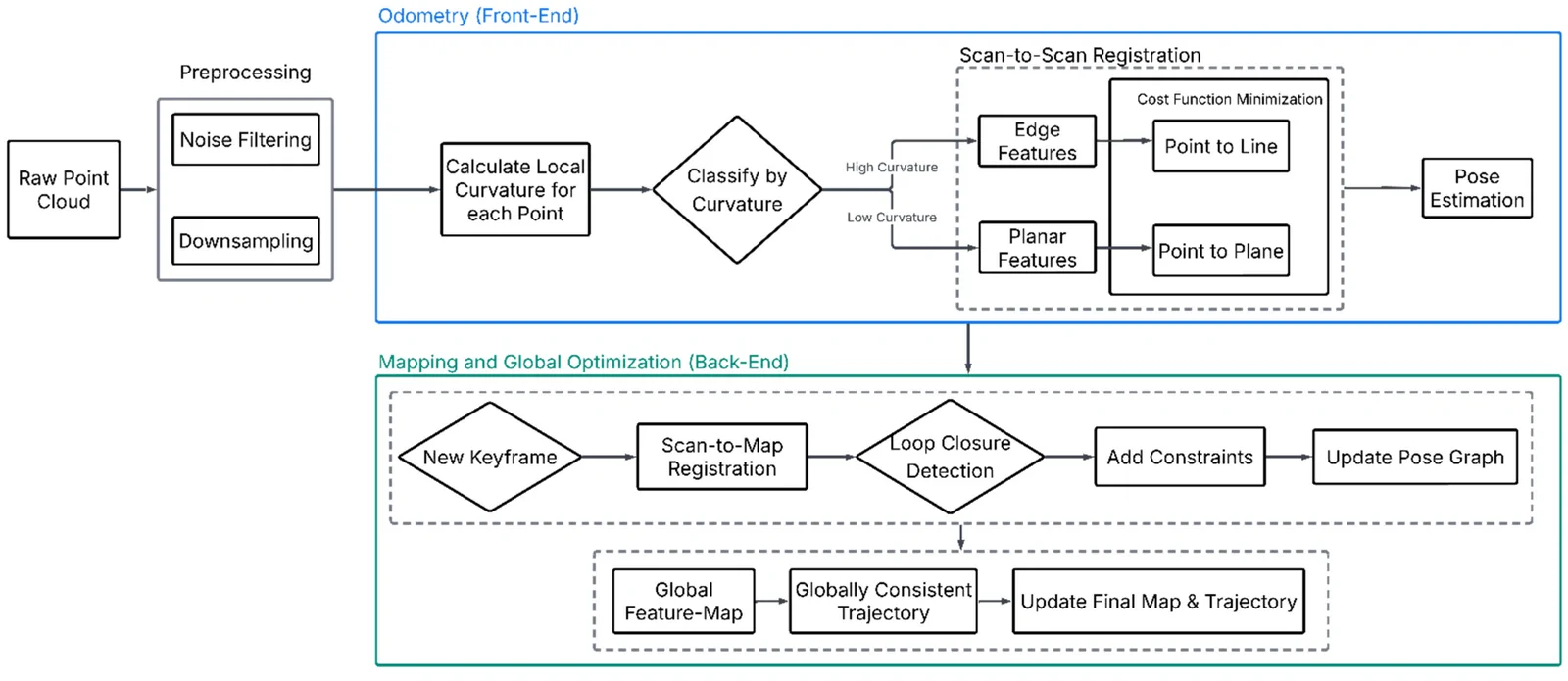
Illustration of a feature-based SLAM pipeline inspired by the architectural principles of LOAM [17]. It highlights the division between high-frequency odometry and low-frequency mapping, as well as key modules, including curvature-based feature extraction, scan registration, and pose graph optimization.

**Figure 5:**
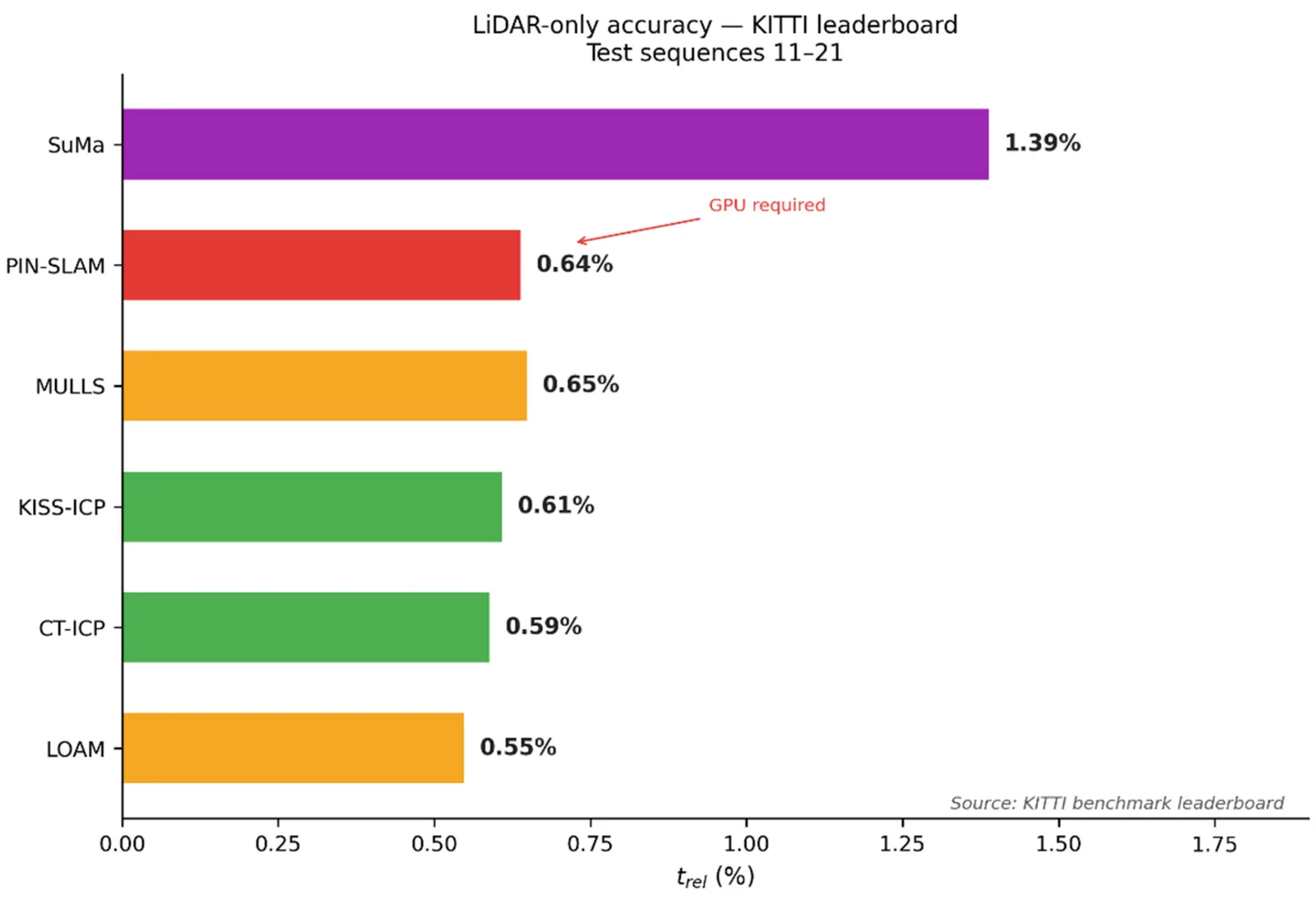
Relative translational errors ($t_{\mathrm{rel}}$) of selected LiDAR-only methods on KITTI odometry benchmark test sequences 11–21. The methods represent feature-based, direct, surfel-based, and neural-implicit paradigms. Values were obtained from the KITTI odometry leaderboard [80] and use the same test sequences and error metric. The translational-error values are therefore directly comparable. Hardware and runtime conditions were not standardized and are not compared.

**Figure 6:**
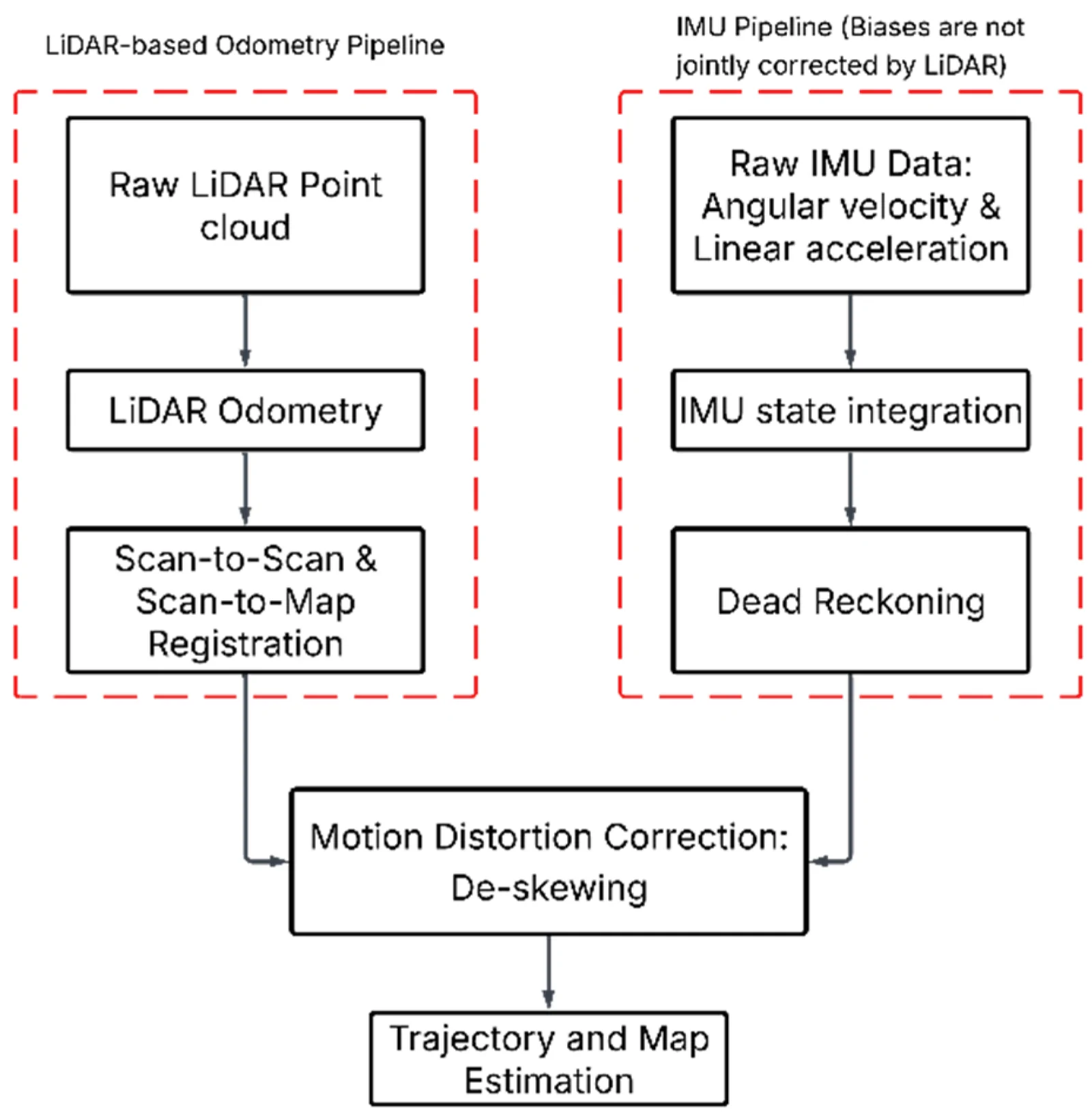
Basic representation of loosely coupled LiDAR-Inertial workflows.

**Figure 7:**
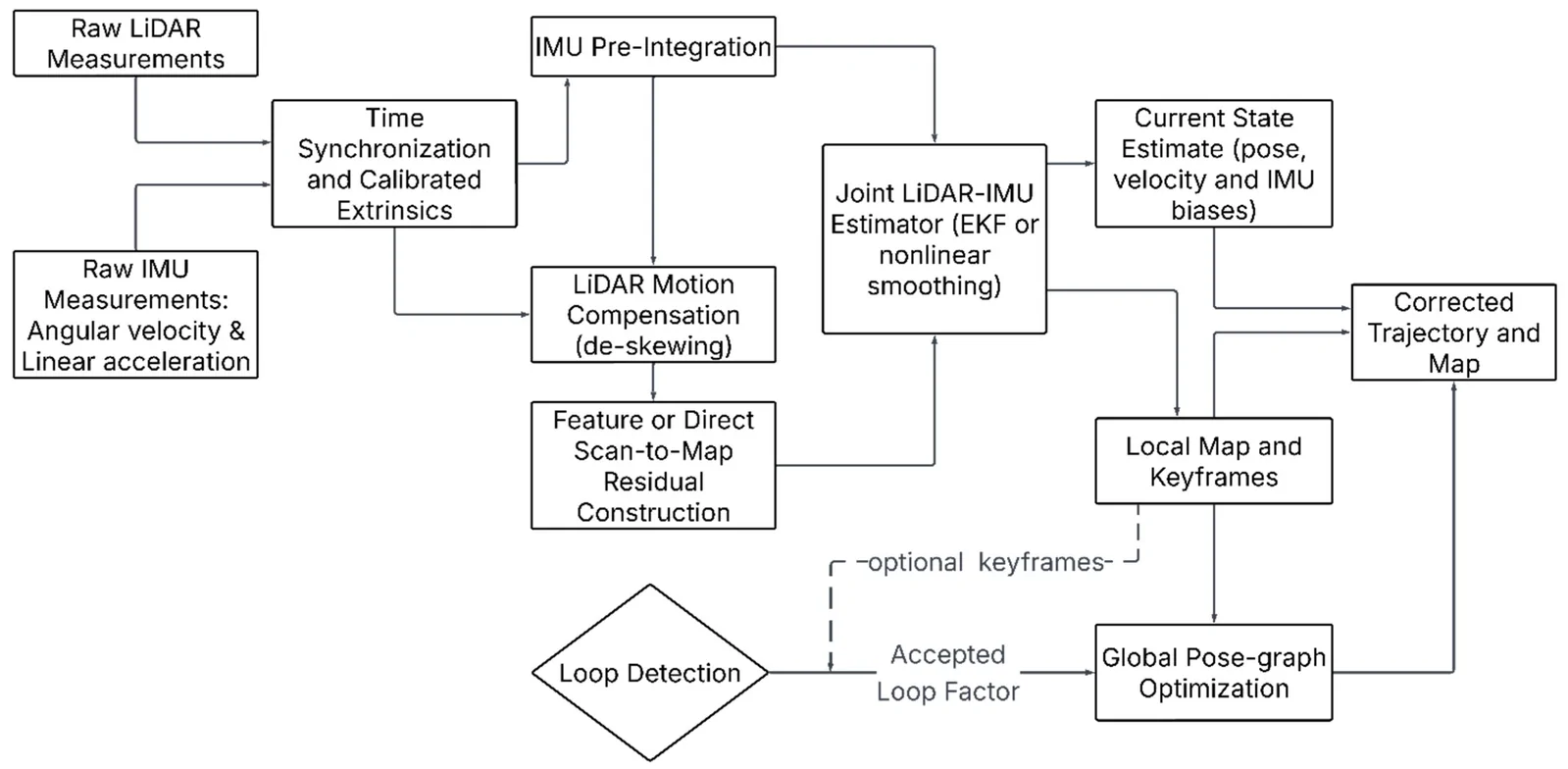
General tightly coupled LiDAR-inertial workflow. Time-aligned LiDAR and IMU measurements jointly contribute to recursive filtering or nonlinear smoothing. An optional keyframe-based back-end verifies loop closures and performs global pose-graph correction.

**Figure 8:**
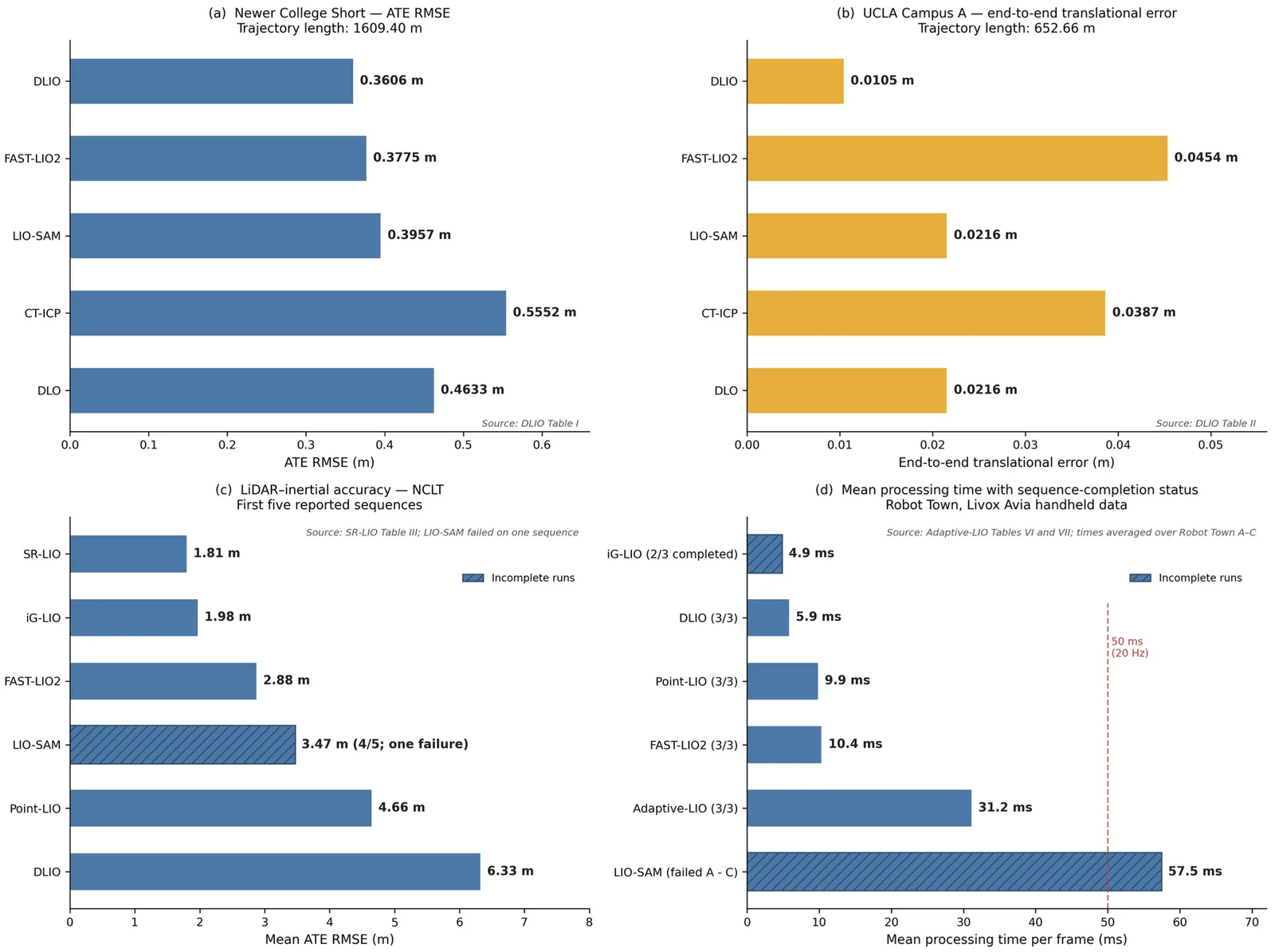
Source-conditioned comparisons of representative LiDAR-only and LiDAR-inertial systems. Results are comparable only within each dataset and metric panel. No ranking is made across panels. (**a**) ATE RMSE on Newer College Short and (**b**) end-to-end translational error on UCLA Campus A, as reported by DLIO [115]. (**c**) Mean ATE RMSE over the first five NCLT sequences, reported from SR-LIO Table III [110]. (**d**) Mean per-frame processing time over Robot Town sequences A–C, as reported by Adaptive-LIO [86]. Hatched bars denote incomplete runs.

**Table 1:** Comparison of the scope, organizing principles, level of architectural detail, and decision-support capabilities of representative LiDAR SLAM reviews and the present review.

Review	Publication Year	Primary Organizing Principle	LiDAR-Only and LiDAR-Inertial Coverage	Architectural/Coupling Granularity	Computational, Robustness, and Deployment Synthesis
Zhu et al. [5]	2024	Camera, LiDAR, and IMU multi-sensor fusion	LIO is one multimodal family; LiDAR-only is not a principal branch	Loose/tight coupling; state-estimator formulations	Emphasizes multimodal-fusion trade-offs; LiDAR-only/LIO cross-branch synthesis is not its focus.
Zhang et al. [6]	2024	Multi-sensor fusion frameworks, methods, and used benchmarks	LiDAR-only versus LiDAR-visual and LiDAR-inertial-visual approaches	Broad coverage of Feature/projection methods; LiDAR-inertial/LiDAR-visual/LiDAR-inertial-visual; loose/tightly coupling	Broad coverage; less explicit cross-branch guidance for system selection.
Xu et al. [9]	2022	Fused-sensor composition plus loose/tight coupling	Primarily multi-sensor LiDAR systems	Strong coupling emphasis	Fusion-oriented analysis; LiDAR-only and cross-branch synthesis are secondary.
Yue et al. [14]	2024	LiDAR forms and configurations: 2D, 3D, and spinning-actuated	LiDAR-only and selected LiDAR-IMU systems	Filter/graph distinctions; coupling is not the primary organizing axis	Emphasizes sensor configurations and applications; coupling-oriented synthesis is secondary.
This review	2026	Dual-axis: sensing configuration and branch-specific architectural organization	LiDAR-only and LiDAR-IMU in one framework	Feature/direct/learning paradigms; loose/tight filter/observer/graph/window structures; shared registration and map analysis	Coherently links computation, failure modes, correction capability, benchmark context, and deployment suitability across both LiDAR-only and LiDAR-inertial branches

**Table 2:** Architectural comparison of front-end scan-registration paradigms, including their registration principles, dominant computational operations, map representations, and typical failure modes.

Registration Paradigm	Core Methodology	Representative Methods	Primary Front-End Computation	Typical Strengths & Failure Modes	Map Representation	Appendix Reference
**Feature-Based (Geometric Primitives)**	Explicit extraction and alignment of edge and planar primitives using point-to-line and point-to-plane residuals.	LOAM, E-LOAM, CSS-LOAM	Feature extraction, curvature estimation, feature association, and residual optimization.	Computationally efficient when reliable features are available; becomes weakly constrained when primitives are sparse, unevenly distributed, or geometrically repetitive.	Sparse feature maps, commonly indexed using k-d trees.	Table A1
	Classification of points into structural classes followed by multi-metric registration.	MULLS	Structural classification, correspondence construction, and multi-metric least-squares optimization.	Exploits varied urban structures but depends on reliable point classification and sufficient representation of each structural class.	Class-labeled structural point maps.	
**Direct Methods**	Scan-to-scan or scan-to-map alignment using point-to-point, point-to-plane, GICP, or related geometric residuals without a discrete feature-selection stage.	DLO, KISS-ICP, LOCUS 2.0	Downsampling, correspondence search, residual construction, local-map updating, and spatial indexing.	Uses a larger portion of the measured geometry; remains vulnerable to motion distortion, poor initialization, and geometrically degenerate scenes.	Point-based or voxelized local maps; implementation-dependent.	Table A2
	Joint estimation of intra-scan motion and geometric alignment using a time-parameterized trajectory.	CT-ICP, Traj-LO	Trajectory interpolation, timestamped residual evaluation, correspondence search, and multi-state optimization.	Reduces rigid-scan motion distortion but introduces additional trajectory states and optimization cost.	Time-associated scans and local submaps.	
	Projective association between incoming scans and rendered surfel maps.	SuMa	Surfel rendering, projective association, normal-based residual construction, and map maintenance.	Efficient scan-to-model association and tolerance of short-lived measurements; depends on rendering quality and stable surface observations.	Surfels containing position, normal, radius, and stability information.	
**Learning-Based/Deep Neural**	Learned point cloud features and pose regression or correspondence estimation.	LO-Net, PWCLO-Net	Neural feature extraction and inference, cost-volume construction, warping, and pose regression.	Can learn complex geometric associations but remains sensitive to training distribution, domain shift, and GPU requirements.	Learned feature representations; persistent mapping support is method-dependent.	Table A3
	Scan-to-implicit-field alignment with simultaneous online map optimization.	PIN-SLAM	Neural-field querying, spatial feature retrieval, scan-to-SDF residual evaluation, and online parameter optimization.	Provides a continuous and compact surface representation but depends on initialization, optimization convergence, and GPU-supported computation.	Sparse neural points supporting a continuous signed-distance field.	

**Table 3:** Taxonomic synthesis and macro-architectural trade-offs of LiDAR-inertial coupling paradigms, illustrating optimization strategies, computational bottlenecks, and global consistency boundaries.

Coupling Paradigm	Representative Systems	State Optimization Strategy	Estimation and Computation Cost	Loop Closure & Global Refinement	Degeneracy & Failure Modes	Appendix Reference
**Loosely Coupled**	LeGO-LOAM, AleGO-LOAM, Adaptive-LIO	A LiDAR-dominant estimator uses IMU information for initialization, de-skewing, ground processing, or fault-aware mode switching without jointly updating LiDAR residuals and inertial states.	Dominated by LiDAR feature extraction, registration, and map maintenance. Cost remains implementation-dependent.	Loop closure is independent of the local estimator and normally requires a separate place-recognition and pose-graph back-end.	LiDAR updates do not directly refine IMU biases. Performance remains sensitive to LiDAR degeneracy, inertial initialization, motion compensation, and sensor faults.	Table A4
**Tightly Coupled (Filter-Based)**	FAST-LIO2, Faster-LIO, iG-LIO, Point-LIO	IMU propagation and LiDAR residuals jointly update pose, velocity, biases, and supported calibration states through recursive filters or incremental state updates.	The active navigation state remains bounded, while correspondence search, residual construction, local-map updating, and data-structure maintenance dominate computation.	The local estimator does not normally retain the complete trajectory. Global correction requires an attached pose graph or smoothing back-end.	Supports high-rate local tracking but may accumulate drift without global constraints. Sensitivity to calibration, timing, map structure, correspondence errors, and geometric degeneracy remains method-dependent.	Table A5
**Tightly Coupled (Graph-Based)**	LIO-SAM, CLINS, Wildcat	LiDAR and inertial constraints are optimized over keyframes, sliding-window states, continuous-time trajectories, or pose-graph variables.	Computation cost grows with the active states, residuals, and factors and is controlled through keyframe selection, marginalization, fixed-lag windows, or staged optimization.	Retained states can be revised using delayed loop-closure, GNSS, or other global factors when the implementation provides the corresponding back-end. Loop closure is not universal.	Enables retrospective or global correction but increases optimization cost and tail latency. Incorrect loop constraints and poorly selected keyframes or window sizes can degrade the solution.	Table A6

**Table 4:** Application-oriented datasets and evaluation evidence relevant to LiDAR and LiDAR-inertial SLAM.

Application Scenario	Representative Datasets or Evaluation Evidence	Dominant Challenge	Typical Validation Purpose	Remaining Validation Gap
**Urban driving and GNSS-degraded urban canyons**	KITTI [80], UrbanNav [168], UrbanLoco [169]	Dynamic traffic, urban canyons, tunnels, GNSS obstruction, multipath, and limited geometric diversity	Odometry accuracy, mapping, and urban localization	Limited repeated long-term evaluation, non-vehicle 6-DoF motion, and explicit failure-and-recovery analysis during prolonged GNSS degradation
**Long-term and multi-season operation**	Oxford RobotCar [170], MulRan [81], EU Long-Term [171], 4Seasons [172], NCLT [83]	Repeated traversals and changes in weather, season, traffic, and scene structure	Place recognition, relocalization, and temporal robustness	Limited harmonized LiDAR/LIO evaluation across repeated traversals
**Aerial, handheld, and mixed indoor–outdoor motion**	NTU VIRAL [173], Newer College [82], M2DGR [174], EuRoC [175]	Platform-specific or aggressive 6-DoF motion, de-skewing, synchronization, and environmental transitions	Inertial coupling, motion compensation, and local mapping	Limited long-duration evaluation and standardized reporting of computational, memory use, and resource budgets
**Crowded indoor and prior map localization**	NAVER LABS localization datasets [176]	Crowds, occlusion, repetitive or textureless structure, low light, and temporal change	Prior-map localization, place recognition, and map alignment	Primarily standardized for visual localization. Limited evaluation protocols for LiDAR/LIO relocalization after tracking failure or controlled map updating across repeated captures
**Adverse weather and natural terrain**	Boreas-RT [177], FoMo [178]	Difficult weather, snow, vegetation and canopy change, and weak or repetitive geometry	Cross-season robustness and sensor complementarity	Few shared LiDAR/LIO baselines spanning road and forest conditions, with limited failure-and-recovery reporting
**Industrial and construction environments**	Hilti SLAM Challenge [179]	Feature-poor or repetitive geometry, varying illumination, and indoor–outdoor transitions	Mapping accuracy, trajectory estimation, loop closure, and robustness	Limited site diversity and long-term evaluation of structural change and resource use.
**Underground and subterranean environments**	DARPA Subterranean Challenge evaluations represented by LOCUS 2.0 [58] and LAMP 2.0 [147]	Darkness, dust, tunnels, geometric degeneracy, and communication constraints	Robust localization, collaborative mapping, and global consistency	Complete multi-robot field trials remain difficult to reproduce, while continuous trajectory ground truth and common communication/resource metrics remain limited

**Table 5:** Synthesis of the principal research directions, unresolved technical problems, and minimum validation evidence for trustworthy, adaptive, and lifelong LiDAR SLAM.

Roadmap Dimension	Findings from the Review	Proposed Research Trajectory	Central Unresolved Problem	Validation Evidence
**Trustworthy estimation and autonomous recovery**	Estimators can remain plausible but incorrect during calibration change, degeneracy, sensor faults, or corrupted measurements.	Integrate calibration-health monitoring, end-to-end uncertainty, diagnosis, measurement gating, fallback selection, and verified recovery.	Determine the cause of degradation, select a safe fallback, and establish when normal operation can resume without oscillation or premature recovery.	Calibration perturbation; NEES/NIS or other consistency tests; detection precision/recall and false-alarm rate; diagnosis accuracy; lead time; recovery time and success; error accumulated during recovery.
**Generalization across environments and sensor conditions**	Fixed configurations degrade during geometric, environmental, and sensor-condition transitions.	Use observable runtime evidence to adapt features, correspondences, losses, map resolution, keyframes, and modality contribution under stability constraints.	Distinguish environmental change from estimator failure and adapt without delayed response, unstable switching, or overfitting to temporary conditions.	Within-sequence transition tests; cross-dataset and cross-platform evaluation; adaptation delay; switch frequency; false adaptations; transition-specific failure rate; adverse-weather and partial sensor loss tests.
**Persistent world models and lifelong map maintenance**	Single-session maps become stale and may confuse transient structure, persistent change, and registration error.	Develop uncertainty-aware multi-session association, temporal state, map versioning, controlled revision, retention, and bounded forgetting.	Decide when observed differences represent genuine change and update the map without deleting useful history or incorporating estimation errors.	Cross-season and long-gap relocalization; positive/negative change precision and recall; false update rate; stale-map failures; version consistency; rollback success; memory growth across sessions.
**Resource-bounded and collaborative operation**	Per-frame efficiency does not prevent cumulative growth in maps, optimization, energy use, or inter-robot communication.	Couple bounded-memory mapping, selective marginalization, hierarchical optimization, compression, and communication-aware submap exchange.	Preserve map utility, relocalization information, and distributed consistency under fixed computation, memory, energy, and bandwidth budgets.	Mission length, area, session, and scaling; runtime distribution and latency; peak memory; energy consumption; communication volume; delay and packet-loss sensitivity; accuracy after compression or marginalization.
**SLAM-planning interface for autonomous navigation**	Planning commonly consumes pose and map estimates without explicit information about confidence, validity, map freshness, or estimator recovery, although planned motion also affects future observability.	Develop bidirectional interfaces that transfer uncertainty, degeneracy, map-validity, and failure status to planning while selecting feasible motions that preserve localization and mapping quality.	Determine how estimation uncertainty, map changes, and localization failures should alter motion decisions without causing unsafe behavior, excessive conservatism, or unstable replanning.	Joint localization and navigation evaluation; task-completion and collision rates; path length and execution time; curvature or dynamic feasibility; uncertainty along the executed path; replanning and recovery latency; computational overhead.

**Note**: NEES = normalized estimation error squared, NIS = normalized innovation squared.

## Data Availability

The data supporting this review are available in the cited publications and publicly accessible benchmark sources referenced in the article. The synthesized comparisons are contained within the article and its appendix tables. No new datasets were generated during this study.

## Appendix A

**Table A1:** Summary of feature-based registration methods for 3D LiDAR SLAM.

Method	Reference	Strengths	Limitations
LOAM	Zhang & Singh, 2014	Pioneering two-threaded feature-based SLAM with edge and planar features. Efficient, modular, and real-time.	Sensitive to feature-degenerate scenes. Lacks loop closure.
A-LOAM	Cao & Qin, 2019	Clean LOAM-derived modular implementation. Uses Eigen and Ceres solver to simplify the code structure. Widely used as an open-source LOAM baseline.	No IMU fusion. No joint optimization, loop closure or global correction.
E-LOAM	Guo et al., 2023	Enhances feature quality using local NDT and LiDAR intensity. Robust in low-structure scenes.	Higher computational complexity.
CSS-LOAM	Gonzalez & Adams, 2019	Multi-scale curvature evaluation for stable feature extraction.	Sensitive to feature extraction parameter tuning. Lacks back-end refinement.
Mesh-LOAM	Y. Zhu et al., 2024	Real-time performance via GPU-accelerated IMLS reconstruction. Scalable memory management with a voxel deletion scheme.	High dependence on a powerful GPU and significant memory usage. High implementation complexity. No loop closure for global drift correction.
MULLS	Pan et al., 2021	Multi-metric optimization over structural classes. Strong accuracy and real-time performance across diverse LiDAR datasets.	Complex pipeline. Requires accurate structural feature classification.
LiODOM	Garcia-Fidalgo et al., 2022	Lightweight LiDAR-only odometry. Efficient local-map data association using weighted point-to-line correspondences with an adaptive local map.	It is an odometry system rather than a full SLAM system with loop closure. Performance depends on reliable line correspondences and local-map quality.
GICP-LOAM	X. Li et al., 2022	Improves LOAM’s registration using voxelized GICP on feature-point clouds. Efficient due to voxelization and multi-threading. IMU-free.	Lacks loop closure and global consistency. Still depends on extracted geometric features.
LOAM_livox	Lin & Zhang, 2020	Tailored to Livox solid-state LiDARs with small FoV. Robust to motion blur.	Limited to small-FoV solid-state LiDAR characteristics.
M-LOAM	Jiao et al., 2021	Online extrinsic calibration without manual intervention. Uncertainty-aware mapping improves robustness.	Requires manually pre-set calibration thresholds. Simple geometric features struggle in degenerate environments.
LOL	Rozenberszki & Majdik, 2020	Segment-based relocalization against a prebuilt 3D point cloud map. Low drift.	Requires an available and reliable prior map. Relocalization quality depends on successful segment matching.
LiDAR Intensity Image-based SLAM	Du & Beltrame, 2023	Uses intensity image and ORB keypoints for scan registration. Handles texture-poor scenes.	Limited feature set. Performance depends on sufficiently distinctive and repeatable LiDAR-intensity features.
T-LOAM	Zhou et al., 2022	Uses TLS-based optimization to reduce outlier influence. High robustness.	Computationally more complex than standard LOAM.
HBA Framework	Liu et al., 2023	Hierarchical BA with a multi-layer graph to improve global consistency while supporting scalability.	High memory and computing demand.
Light-LOAM	Yi et al., 2024	Stable feature selection and geometric voting. Lightweight and suitable for resource-constrained platforms.	Graph-based matching adds correspondence-selection complexity. Robustness still depends on sufficient stable feature samples.
Dynamic-Aware Odometry	Huang et al., 2024	Joint dynamic weighting and pose estimation. Strong in dynamic scenes.	Requires semantic/motion cues. Increased complexity.

**Table A2:** Summary of direct registration LiDAR SLAM methods.

Method	Reference	Strengths	Limitations
ICP	Besl & McKay, 1992	Fundamental registration baseline. Simple and widely adopted.	Requires good initialization. Sensitive to noise, outliers, and local minima.
G-ICP	Segal et al., 2009	Models local surface structure. Probabilistic registration framework. Often more accurate than point-to-point ICP.	Higher computational cost. Requires local covariance estimation.
KISS-ICP	Vizzo et al., 2023	Minimalist LiDAR-only odometry. Real-time capable. Competitive accuracy with simple components.	No loop closure or global optimization. Still inherits ICP sensitivity to degeneracy.
MAD-ICP	Ferrari et al., 2024	Uncertainty-aware scan integration. Robust PCA-based kd-tree registration	More complex data association/map management. Depends on reliable uncertainty estimation.
Fast-ICP	J. Zhang et al., 2021	A majorization–minimization formulation and Anderson acceleration improve convergence, while a Welsch-function metric improves registration robustness. Faster convergence than standard ICP.	Still a pairwise registration method. Sensitive to initialization and overlap quality.
CT-ICP	Dellenbach et al., 2022	Handles intra-scan distortion. LiDAR-only continuous-time ICP. Includes loop closure extension.	Higher runtime than simpler ICP odometry. Depends on timestamp accuracy and trajectory interpolation.
HDL-Graph	Koide et al., 2019	Modular graph-based SLAM framework. Supports G-ICP/NDT registration. Includes loop closure/global correction.	Heavier than pure odometry pipelines. Parameter tuning required.
RTAB-Map (LiDAR-mode)	Labbé & Michaud, 2019	Versatile method with loop closure enabled. Graph optimization supported.	Higher memory/compute management complexity. Performance depends on configuration and loop-closure reliability.
LOCUS 2.0	Reinke et al., 2022	Computationally efficient LiDAR odometry. Memory-bounded sliding-window map. Robust in complex underground environments.	Odometry only; lacks global optimization. Sliding-window mapping trades map extent for bounded memory.
Cartographer (LiDAR-only mode)	Hess et al., 2016	Submap-based scan matching. Real-time loop closure. Pose-graph optimization.	Many tuning parameters. 2D LiDAR focus in the cited work. Can be computationally demanding.
ART-SLAM	Frosi & Matteucci, 2022	Loop closure and pose-graph optimization. Full 6-DoF LiDAR SLAM.	More complex architecture. Performance depends on loop detection and initialization quality.
DLO	K. Chen, Lopez, et al., 2022	Real-time G-ICP odometry. Designed for perceptually degraded environments.	No loop closure/global optimization. Odometry drift can accumulate over long trajectories.
NDT-LOAM	S. Chen, Ma, et al., 2022	Weighted NDT registration. LiDAR-only odometry and mapping. Robustness in noisy/unstructured scenes.	Sensitive to voxel/grid parameters. Limited global optimization.
ERASOR	Lim et al., 2021	Learning-free dynamic object removal. Useful for static map building.	Requires accumulated map/scan evidence. Not an odometry method by itself.
SuMa	Behley & Stachniss, 2018	Uses surfel map and projective data association for efficient scan-to-model alignment. Robust to transient/dynamic measurements.	Requires accurate surfel rendering. Dynamic objects can still corrupt the map if they form stable surfels over consecutive scans.
IMLS-SLAM	Deschaud, 2018	Scan-to-model registration. High mapping precision using a continuous implicit surface model.	Computationally heavier than sparse feature methods. No explicit loop closure in the core method.
SLAMesh	Ruan et al., 2023	CPU-only real-time mesh mapping. Robust point-to-mesh alignment.	Mesh management overhead. The paper does not describe explicit point-wise motion compensation, so robustness under rapid non-uniform motion remains uncertain.
Puma-LO	Vizzo et al., 2021	High-fidelity mesh mapping using Poisson surface reconstruction. Ray-casting-based data association.	Not real-time. Sliding window meshing introduces latency.
MD-SLAM	Di Giammarino et al., 2022	Direct multi-cue alignment. Uses LiDAR-derived range/intensity/normal images. Graph-based SLAM formulation.	Sensitive to projected image quality. Direct alignment depends on sufficient range/intensity structure.
ECTLO	Zheng & Zhu, 2023	Designed for prism-based small-FoV LiDAR. Compact range-image representation. Continuous-time filtering.	Specialized for non-repetitive small-FoV sensors. May require adaptation for other LiDAR scanning patterns.
SROM	Rufus et al., 2020	Coarse-to-fine registration. Robust initialization with POC followed by ICP refinement.	Depends on reliable POC initialization. Less general than full SLAM systems with loop closure.
Traj-LO	Zheng & Zhu, 2024	IMU-free continuous-time LiDAR odometry. Handles motion distortion.	Optimization overhead. Sensitive to smoothness/segment settings.
I2EKF-LO	Yu et al., 2024	Dual-iteration EKF for LiDAR-only odometry allows for iterative refinement of motion-distortion correction and point-to-plane updates. Dynamically adjusts process noise for motion robustness.	Odometry-only method. Uses a motion model rather than IMU propagation. No loop closure or global optimization.

**Table A3:** Summary of learning-based and semantic SLAM approaches.

Method	Reference	Type	Training	Dataset	GPU/Inference Time	Strength	Limitations
LO-Net	Li et al., 2019	Supervised DL odometry	Supervised using KITTI ground-truth poses	KITTI; Ford Campus Vision and LiDAR	NVIDIA 1080 Ti + Intel i7 CPU; 80.1 ms/scan total	Early end-to-end neural LiDAR odometry. Mask-weighted loss reduces influence of dynamic/unreliable regions.	Requires ground-truth training data. Encodes point clouds into structured matrices. No loop closure.
DeepVCP	Lu et al., 2019	Supervised DL registration	Supervised using ground-truth poses	KITTI; Apollo-SouthBay	GTX 1080 Ti + Core i7-9700K; about 2 s/frame pair	Learns keypoints and virtual correspondences. Robust to inaccurate initial poses.	Not real-time. Registration module rather than full odometry/SLAM.
SLOAM	Chen et al., 2020	Semantic LiDAR odometry/mapping	Supervised semantic segmentation using custom labeled forest scans	Custom UAV and handheld forest datasets	Not reported in paper	Semantic tree-cylinder landmarks improve forest odometry and enable DBH estimation.	Domain-specific to forest/tree landmarks. Depends on segmentation and landmark extraction quality.
PWCLO-Net	Wang et al., 2021	Supervised DL odometry	Supervised using KITTI ground-truth poses	KITTI; 00–06 train, 07–10 test	NVIDIA RTX 2080Ti; runtime not clearly reported	Hierarchical pyramid-warping-cost volume refinement. Trainable mask filters unreliable/outlier motion patterns.	Odometry only. No loop closure or global map optimization.
ELiOT	Lee et al., 2025	Supervised DL odometry	Supervised; KITTI plus simulated/digital-twin data	KITTI; CARLA/digital-twin evaluations	High-end NVIDIA GPU with 48 GB VRAM; runtime not clearly reported	Transformer-based feature aggregation. Avoids 3D-to-2D projection. Explores real/sim/digital-twin training.	KITTI error remains higher than several geometric baselines. High GPU requirement.
SuMa++	Chen et al., 2019	Semantic-aided LiDAR SLAM	Uses pre-trained RangeNet++ semantic segmentation	KITTI road; KITTI odometry	NVIDIA Quadro P4000; 75 ms segmentation + 48 ms surfel mapping	Semantic labels improve ICP robustness and reduce dynamic-object map corruption. KITTI road average translational error improves from 1.35% for SuMa to 1.04%.	Cannot filter dynamic objects in the first observation. Depends on semantic segmentation quality.
SHINE-Mapping	Zhong et al., 2023	Implicit neural mapping	Scene-specific optimization with known poses	MaiCity; Newer College; KITTI example	Hardware/runtime not clearly reported in paper	Sparse hierarchical implicit representation. Strong Chamfer-L1 results. Reduces catastrophic forgetting.	Requires known poses. Mapping only, not full SLAM. No loop closure.
LocNDF	Wiesmann et al., 2023	Neural distance field localization	Scene-specific offline optimization	Apollo-SouthBay; indoor MCL experiment	NVIDIA RTX A5000; MCL about 2.6 Hz; training about 20 min per key pose	Compact neural distance field supports scan registration and Monte Carlo localization.	Offline map training. Not online/incremental SLAM.
LONER	Isaacson et al., 2023	Neural implicit LiDAR SLAM	Online scene-specific optimization	Fusion Portable; Newer College	NVIDIA A6000; 14 ms tracking; about 18 Hz map update	First real-time LiDAR-only neural implicit SLAM, as stated by the authors. Real-time tracking and mapping.	Scalability to city-scale remains limited. No inertial data, which could improve rapid rotation and feature-sparse tracking
INF-SLiM	Zhang et al., 2025	Semantic implicit neural mapping	Self-supervised geometry + semantic/panoptic supervision	SemanticKITTI; SemanticPOSS	NVIDIA RTX 4090; about 3.8 s/frame in reported timing table	Joint geometry, semantic, and panoptic implicit mapping. Large-scale outdoor semantic reconstruction.	Requires input/ground-truth poses. No integrated odometry or loop closure. Below real-time.
PIN-SLAM	Pan et al., 2024	Neural implicit SLAM	No task-specific offline pre-training; online optimization	KITTI; MaiCity; Newer College; Hilti-21	NVIDIA A4000; 0.14 s/frame full system, 7.1 Hz; light version 11.3 Hz	Point-based implicit map. Scan-to-SDF odometry. Loop closure and pose graph optimization. Compact globally consistent mapping.	Requires GPU for reported real-time operation. Online neural optimization adds complexity. Full version below 10 Hz LiDAR frame rate.

**Table A4:** Summary of loosely coupled systems.

Method	Reference	Strengths	Limitations
LeGO-LOAM	Shan & Englot, 2018	Lightweight and optimized for ground robots. Ground segmentation reduces unreliable features. Two-step LM optimization improves efficiency.	IMU is used only as an initial pose guess in the reported experiments. No joint LiDAR-IMU state or bias estimation is described. Ground-optimized design may require changes for non-ground platforms.
LeGO-LOAM + Scan Context	Shan & Englot, 2018; Kim & Kim, 2018	Scan Context improves loop closure/place recognition. Can improve global consistency when added to a LeGO-LOAM-style front-end.	Scan Context improves loop detection, not LiDAR-IMU state estimation. No IMU bias optimization is introduced by Scan Context.
Adaptive LeGO-LOAM	S. W. Lee et al., 2019	Adaptive/fast feature extraction for real-time SLAM.	Does not optimize or correct IMU state. Limited robustness in degenerate scenes.
Adaptive-LIO	Zhao et al., 2025	Dynamically adapts to observability and scene complexity. Mitigates IMU faults and saturation through adaptive switching between LiDAR–inertial and LiDAR-only odometry.	Complex control logic. Performance may vary with environment predictability.
LOFF	Zhang et al., 2024	Direction-separated fusion compensates LiDAR degeneracy using optical-flow odometry.	Requires optical-flow sensing. Evaluated mainly in tunnel/GNSS-denied degeneracy scenarios.

**Table A5:** Summary of filter-based tightly coupled SLAM methods.

Method	Reference	Strengths	Limitations
FAST-LIO	Xu & Zhang, 2021	Better scalability using a novel Kalman Gain. Highly computationally efficient. Ideal for resource-constrained machines.	Feature-based operation can degrade when reliable geometric features are sparse. No integrated loop-closure/global optimization back-end.
FAST-LIO2	Xu et al., 2022	Direct raw-point registration without hand-crafted feature extraction. Adaptable to different LiDAR scanning patterns. Efficient incremental mapping using ikd-tree.	No integrated global loop-closure optimization. Still depends on reliable scan-to-map correspondence quality.
Faster-LIO	Bai et al., 2022	Fast sparse incremental voxel map using iVox. Avoids repeated tree maintenance costs. Lightweight and real-time oriented.	Approximate voxel-based neighbour search can trade accuracy for speed. No integrated loop-closure/global optimization.
Eq-LIO	Tao et al., 2025	Equivariant filter improves consistency and robustness. Fixed-origin linearization helps suppress linearization error. Supports gravity constraint and extrinsic calibration.	Mathematically and implementation-wise, more complex. No explicit loop-closure/global optimization module.
Point-LIO	He et al., 2023	Point-by-point update reduces in-frame motion distortion. High-frequency odometry output. Robust to IMU saturation through stochastic-process IMU modeling.	Implementation complexity due to stochastic modeling. No integrated loop-closure/global optimization.
SE(3)-LIO	Shin et al., 2026	Smooth IMU propagation on SE(3). Improved motion compensation under aggressive motion. Robust under sparse measurements.	More complex manifold propagation. Performance still depends on voxel-map/correspondence quality.
MSC-LIO	T. Zhang et al., 2025	Highly efficient data association accelerates processing time. Online LiDAR-IMU time-delay estimation. Demonstrated real-time performance on Jetson AGX Xavier.	State maintenance overhead due to retaining keyframe states in the sliding window. Yaw may remain weakly observable in some conditions.
VoxelMap	Yuan et al., 2022	Probabilistic adaptive voxel mapping. Explicit plane uncertainty modeling. Efficient coarse-to-fine voxel representation.	Primarily a mapping/odometry representation rather than full SLAM. No loop-closure module.
VoxelMap++	C. Wu et al., 2024	Mergeable voxel/plane structure reduces redundancy. Supports LiDAR-only and LiDAR-inertial odometry. Suitable for embedded/resource-constrained platforms.	Plane merging assumes locally planar structure. Performance depends on voxel and merge criteria.
R-VoxelMap	Xi et al., 2026	Recursive RANSAC plane fitting improves plane validity. Reuses outlier points through deeper octree subdivision. Reduces erroneous merging of different physical planes.	Acts more as a voxel-mapping extension for online LiDAR odometry, not a complete tightly coupled LIO system. Recursive fitting and validity checking add map-management complexity.
GenZ-LIO	D. Lee et al., 2026	Generalizable across indoor–outdoor scale transitions. Scale-aware adaptive voxelization. Hybrid point-to-plane/point-to-point residuals mitigate degeneracy. Voxel-pruned correspondence search reduces computation.	Odometry-only framework without loop closure. Long-term drift can accumulate in extended trajectories.
iG-LIO	Z. Chen et al., 2024	Voxel-based GICP with surface covariance estimation. Efficient and accurate in dense scenes. Incremental GICP tightly coupled with IMU constraints. Voxel-based surface covariance estimation. Fast because of O(1) hash-index query for voxel lookup.	Voxel covariance and map management increase implementation complexity. No integrated loop-closure/global optimization.
LOG-LIO	K. Huang, Zhao, Zhu, et al., 2023	Ring FALS efficiently estimates normals from LiDAR ring structure. Hierarchical point-to-surfel association. Improves local geometric information use.	Designed around ring-structured spinning LiDAR. Simplified uncertainty modeling compared with LOG-LIO2. Odometry-only back-end.
LOG-LIO2	K. Huang et al., 2024	Comprehensive uncertainty model includes range, bearing, incident angle, and roughness. LUFA enables efficient uncertainty propagation. Better residual weighting.	More complex uncertainty modeling. Odometry-only framework.
LIO-PPF	X. Chen et al., 2023	Plane pre-fitting and skeleton tracking reduce repeated kNN plane fitting. iPCA improves robustness to noisy/non-strict planes. Lower time and memory cost.	Most effective when sufficient planar structure exists. Less suitable for strongly non-planar scenes.
LIO-GVM	Ji et al., 2024	Gaussian voxel map supports probabilistic registration. Multi-resolution voxel representation. Accurate tightly coupled LIO.	More complex map representation. Memory and management cost can grow in large scenes.
PPLIO	H. Lee et al., 2025	Planar-feature-based MSCKF. Robust plane association under initial pose error. Plane RANSAC removes outliers.	Requires reliable plane extraction. Less suitable where planar features are weak or scarce.
COIN-LIO	Pfreundschuh et al., 2024	Uses LiDAR intensity images to improve degeneracy handling. Photometric residuals complement geometric constraints. Strong in tunnels/flat fields.	Requires informative intensity texture. Adds photometric processing overhead. No global loop-closure back-end.
DLIO	Chen et al., 2023	Lightweight direct LIO. Continuous-time motion correction. Nonlinear geometric observer supports robust state estimation.	No integrated loop-closure/global optimization. Accuracy depends on reliable scan-to-map registration and state initialization.
SR-LIO	Yuan et al., 2024	Sweep reconstruction generates high-frequency pseudo-scans. Reduces temporal sparsity in spinning LiDARs. Improves motion handling.	Designed primarily for spinning LiDARs. Adds sweep segmentation/reconstruction complexity.
SLICT2	T. M. Nguyen et al., 2024	B-spline continuous-time LIO. Fast convergence using solve-associate-solve loop.	Odometry-focused system without large-scale loop closure. Continuous-time formulation adds implementation complexity.
LINS	Qin et al., 2019	Lightweight iterated ESKF-based LIO. Robust real-time navigation in varied scenarios. Supports mapping module.	No full global graph optimization in the odometry estimator. Long-term consistency remains limited without loop closure.
ESKF_LIO	Zhao, 2022	Open-source ESKF-based tightly coupled LIO implementation. Useful engineering baseline.	Repository reference limits verifiability. Detailed peer-reviewed evaluation is not available from the cited source.
Inv-LIO	Shi et al., 2023	Invariant EKF improves consistency on Lie groups. Reduces linearization error. Robust under aggressive motion.	More complex filter formulation. Less broadly validated than FAST-LIO-style baselines.
SW-LIO	Z. Wang, Liu, et al., 2023	Sliding-window tightly coupled LIO. Connects current state with previous keyframes. Adds ground, bias, and gravity residuals.	Higher state/window maintenance cost. No full global loop-closure back-end.
LIO-EKF	Y. Wu et al., 2024	Simple EKF design. Adaptive data-association threshold uses pose uncertainty, voxel discretization, and sensor noise. Low computational load.	Simplified point-to-point front-end may limit performance in complex scenes. No global optimization module.
SRIF-LIO	K. Li, Ouyang, et al., 2021	SRIF framework enables real-time operation on ARM platforms. Designed for autonomous-vehicle localization.	Localization against a prior map only, not full SLAM. Performance depends on prior map quality/currentness.
FMCW-LIO	M. Zhao et al., 2024	Uses Doppler velocity from FMCW LiDAR. Robust in structure-degenerate scenes. Correspondence-free velocity observation improves degeneracy handling.	Requires specialized LiDAR hardware. General adoption limited by sensor availability.
RI-LIO	Zhang et al., 2023	Adds reflectivity-image residuals to geometric IEKF update. Corrected projection model improves reflectivity alignment. Helps in low-geometric-feature scenes.	Additional computation from image alignment. Sensitive to reflectivity texture and image quality.
EKF-LOAM	Junior et al., 2022	Fusion of LeGO-LOAM, IMU, and wheel odometry in EKF. Improves confined/few-feature environment mapping. Useful for ground robots in tunnels/ducts.	Depends on wheel odometry quality and slip conditions. Still inherits LeGO-LOAM feature-front-end limitations.
HD-LIO	Wang et al., 2024	Low-level IEKF plus high-level sliding-window factor graph refinement. Anti-degeneration point-to-distribution constraints. Improves distribution-based LIO robustness.	More complex hierarchical architecture. Added computational cost from factor graph refinement.
Highway Environments-LIO	S. Zhao et al., 2019	Designed for large-scale highway environments. CNN segmentation filters dynamic objects. ESKF-based fusion supports real-time odometry.	Depends on segmentation quality. Designed for highway-like operating conditions.
DAMS-LIO	Han et al., 2023	Degeneration-aware modular fusion. Incorporates external relative pose measurements only when degeneration is detected.	Performance depends on the quality of the external odometry source. No full global optimization back-end.
EVA-LIO	Chen et al., 2026	Targetless LiDAR-motor calibration. Environment-adaptive voxel/map parameter selection. Robust for spinning actuated LiDAR systems.	Performance degrades in highly dynamic scenarios. LM-Calibr relies on local plane extraction and sufficient geometric identifiability.
OMC-SLIO	S. Wang et al., 2023	Online multiple calibration for spinning LiDAR systems. Estimates mechanism and sensor calibration parameters. Addresses spinning LiDAR-IMU calibration online.	Higher-dimensional state increases estimation complexity. Specific to spinning LiDAR platforms.
KN-LIO	Z. Wang et al., 2025	Couples EKF-based state estimation with neural SDF mapping. Produces dense/high-fidelity map reconstruction. Supports online neural-field updates.	Neural mapping increases computation and memory demand. More complex than purely geometric LIO.
R3LIVE	J. Lin & Zhang, 2022	Combines LIO and VIO subsystems in an ESIKF framework. Real-time dense RGB-colored mapping. Visual photometric updates enrich LiDAR-inertial mapping.	Requires calibrated/synchronized LiDAR, IMU, and camera inputs. Multi-modal fusion and dense color mapping increase system complexity.

**Table A6:** Summary of graph-based tightly coupled and related graph/back-end SLAM methods.

Method	Reference	Strengths	Limitations
LIO-SAM	Shan et al., 2020	Factor graph integrates IMU preintegration, LiDAR odometry, GPS factors, and loop closure. Real-time front-end with global consistency through loop closure.	Performance depends on reliable feature extraction and scan matching. Requires parameter tuning for different platforms and environments.
MILIOM	T. M. Nguyen et al., 2021	Fuses multiple lidars with complementary FoVs for enhanced observability and robustness. Tightly coupled, keyframe-based graph optimization.	Requires multiple, well-calibrated lidars, increasing hardware cost and complexity. Synchronization and merging add overhead.
LIO-mapping (LIOM)	Ye et al., 2019	Sliding window optimization with tight lidar-IMU coupling. Robust to diverse motion.	Relies on hand-crafted features. May be sensitive to feature degradation.
D-LIOM	Z. Wang et al., 2023	Uses direct scan-to-submap registration. Avoids sparse handcrafted feature extraction. Provides denser geometric constraints than feature-only methods.	Higher computational demand than sparse feature-based approaches. Performance depends on the quality of probabilistic local submaps.
DMSA-SLAM	Skuddis & Haala, 2024	Dense Multi Scan Adjustment without explicit feature extraction. Robust to limited scan overlap and dynamic-object outliers. Supports sliding-window odometry and large-scale keyframe optimization.	Computationally expensive. Reported processing is slower than real time. Jacobian computation is a major bottleneck.
PA-LVIO	Tang et al., 2026	Pose-only BA avoids maintaining landmark states. Real-time operation on desktop and ARM platforms. Supports RGB-rendered point cloud mapping and online spatial-temporal calibration.	System accuracy is affected by synchronization and dynamic objects. F2M factor effectiveness can vary across scenarios. More complex than LiDAR-only LIO.
Consistency-Improved BA LIO	X. Li et al., 2026	Estimator Consistency: Stereographic projection avoids singularities and spurious information gain. Preserved Observability: MAP and FEJ maintain accurate covariance. Numerical Stability: Bounded partial derivatives stabilize optimization.	Lower Optimization Frequency: High computational load limits back-end joint optimization to at most once every three frames. Reliance on Explicit Features: planar and edge features.
LVI-SAM	Shan et al., 2021	Jointly optimizes visual odometry, LiDAR odometry, IMU preintegration, and loop closure factors. Improves robustness in sensor-degraded scenarios through LiDAR-visual-inertial complementarity.	Requires camera-LiDAR-IMU calibration. Visual subsystem depends on sufficient image quality and trackable visual features.
LIO-Fusion	W. Wu et al., 2023	Robustly fuses multiple sensors (lidar, IMU, GNSS, wheel odometry) with health checks. Excels in degraded scenes.	High system complexity; Requires multiple sensors (wheel encoders, GNSS) to achieve its full potential.
LiLi-OM	K. Li et al., 2021	High-performance and versatile; works on both mechanical and solid-state lidars. Feature extraction is tailored to irregular scan patterns.	Feature-based, can still struggle in feature-poor scenes, especially with small-FoV solid-state lidars.
R-LIO	K. Chen, Zhan, et al., 2022	Specifically designed for rotating lidars, improving mapping coverage.	Hardware-specific. Not generally applicable to standard fixed lidars.
Spin-LOAM	Yan et al., 2023	Designed for spinning actuated lidars. Adaptive scan accumulation improves robustness.	Hardware-specific. Its innovations are less relevant for standard fixed sensors.
CLINS	Lv et al., 2021	Continuous-time B-spline trajectory enabling robust fusion of asynchronous lidar and IMU data. Efficient two-stage global correction.	Computational cost due to using non-rigid registration and B-spline optimization, with Jacobians being a performance bottleneck.
Wildcat SLAM	Ramezani et al., 2022	High Robustness: Proven in challenging environments. Continuous-time & multi-agent by using B-spline odometry for robustness and a submap-based PGO back-end.	Multi-agent operation requires peer-to-peer submap exchange and repeated collective pose-graph optimization, introducing communication and computational overhead.
IN2LAAMA	Gentil et al., 2021	Continuous-time motion model using Gaussian Processes for highly accurate motion distortion correction. Supports targetless online extrinsic autocalibration.	Offline, batch optimization framework. Not designed for real-time operation. High memory consumption.
BALM	Z. Liu & Zhang, 2021	Introduces local bundle adjustment to optimize LOAM-style front-ends, improving local pose refinement and global trajectory smoothness.	Dependent on LOAM features. Less effective in dynamic or poorly structured environments.
KFS-LIO	Li et al., 2021	Key-feature selection algorithm reduces computational cost. Quantitative evaluation of feature quality.	The greedy feature selection process may not yield a globally optimal constraint set. Still reliant on salient features (edge and planar points).
LIO-Vehicle	Xiao et al., 2022	Incorporates vehicle kinematics. Improves odometry on wheeled platforms.	Limited to non-holonomic ground vehicles. Less flexible for aerial robots.
Semantic-LIO	Bu et al., 2022	Uses semantic segmentation to remove moving-object points. Improves robustness in dynamic environments. Reduces misleading constraints from temporary objects.	Depends on segmentation quality. May degrade when dynamic-object classes are missed or misclassified.
Intensity-Augmented- LI	H. Li et al., 2022	Augments geometry with intensity features, making it robust in geometrically degenerate environments. Tailored for solid-state lidars.	Performance relies on the presence of salient intensity features. The line map management adds complexity.
RF-LIO	Qian et al., 2021	Removal-first strategy improves operation in highly dynamic environments. Uses adaptive multi-resolution range images before scan-to-map matching. Does not require learning-based training data.	Depends on reliable moving-object removal. Range-image preprocessing adds system complexity.
S-Graphs+	Bavle et al., 2023	Hierarchical graph with robot poses, walls, rooms, and floors. Combines SLAM with semantic/structural scene understanding. Suitable for multi-floor indoor mapping.	Depends on reliable semantic and structural extraction. More complex than metric-only SLAM graphs.
LIO-SEGMOT	Y. K. Lin et al., 2023	Simultaneously performs SLAM and multi-object tracking. Asynchronous updates provide continuous object trajectories.	Performance is dependent on an external 3D object detector. High system complexity.
RDC-SLAM	Xie et al., 2022	Supports decentralized multi-robot SLAM with robust inter-agent communication.	Requires a stable network and shared observation regions for effective performance.
ColaSLAM	Huang et al., 2021	Edge-assisted SLAM offloads computation to nearby edge nodes and supports collaborative multi-robot map fusion. Supports collaborative multi-agent mapping.	Latency and connectivity limitations may affect real-time performance.
LAMP 2.0	Chang et al., 2022	Robust multi-robot SLAM with GNC and scalable loop closure. Adaptable to diverse platforms. Excels in GPS-denied, large-scale environments.	Centralized design may limit large-team scalability. High system complexity and resource demands for modules like GNN and GNC.
LIPS	Geneva et al., 2018	Plane-based LiDAR-inertial SLAM. Uses singularity-free CP plane parameterization. Effective in structured planar environments.	Depends on sufficient planar structure. Less suitable for highly non-planar or cluttered scenes.
NV-LIO	Chung & Kim, 2024	Uses normal-vector-based registration. Designed for robust SLAM in narrow and multi-floor indoor environments. Includes degeneracy detection and viewpoint-based loop closure.	Requires dense LiDAR scans for reliable normal extraction. Additional normal-vector preprocessing increases computation.
Map-centric SLAM	Park et al., 2022	Maintains a global surfel map instead of a conventional pose graph. Uses elastic map deformation for loop-closure correction. Supports dense continuous-time map-centric SLAM.	Map deformation can accumulate distortion at larger scales. Partial observation remains a limitation.
GLIM	Koide et al., 2024	GPU-accelerated scan-matching factors. Real-time range-inertial localization and mapping. Supports fixed-lag smoothing, keyframe matching, and global matching-cost optimization.	Requires GPU acceleration for intended performance. Global optimization cost grows with map/submap size.
LoLa-SLAM	Karimi et al., 2021	Low-latency 6-DoF LiDAR SLAM for UAVs. Uses continuous scan slicing and concurrent matching. Provides a high update rate for control-oriented localization.	Smaller slice sizes reduce available features and can degrade accuracy. Not a tightly coupled LiDAR-inertial graph optimizer.
FF-LINS	Tang et al., 2023	Designed for solid-state LiDARs with non-repetitive scan patterns. Frame-to-frame registration without explicit geometric feature extraction. Online LiDAR-IMU extrinsic and temporal calibration.	No loop-closure mechanism. Frame-to-frame estimation can accumulate drift. Calibration quality depends on sufficient motion and observability.
LIMOT	Zhu et al., 2024	Joint ego-motion and object tracking. Effective in dynamic environments.	Increased complexity. Requires accurate object detection.

**Table A7:** Quantitative evidence supporting Figure 5 and Figure 8. **Group A** reports selected results from the KITTI odometry benchmark leaderboard for test sequences 11–21, where $t_{\mathrm{rel}}$ and $r_{\mathrm{rel}}$ denote average relative translational and rotational errors, respectively [80]. **Group B** presents selected Newer College Short and UCLA Campus A results reported in the DLIO study [115]. **Group C** presents selected mean ATE RMSE results over the first five NCLT sequences reported in the SR-LIO study, with failed runs retained explicitly [110]. **Group D** presents selected Robot Town end-to-end translation errors, mean processing times, and completion outcomes reported in the Adaptive-LIO study [86]. ATE = absolute trajectory error; RMSE = root-mean-square error; E2E = end-to-end.

**Group A: Benchmark-aligned KITTI trajectory error comparison [80]—Supporting Figure 5. Reported runtime is for reference only.**								
**System**	Category		$t_{\mathrm{rel}}$ (%)	$r_{\mathrm{rel}}$ (°/100 m)	Runtime (ms/frame)	Hardware		
**LOAM** [17]	LiDAR-only, feature-based		0.55	0.13	100	2-core CPU @ 2.5 GHz		
**CT-ICP** [22]	LiDAR-only, direct		0.59	0.14	60	1-core CPU @ 3.5 GHz		
**KISS-ICP** [50]	LiDAR-only, direct		0.61	0.17	50	1-core CPU @ 4.5 GHz		
**MULLS** [40]	LiDAR-only, feature-based		0.65	0.19	80	4-core CPU @ 2.2 GHz		
**PIN-SLAM** [21]	LiDAR-only, learning-based		0.64	0.15	100	GPU @ >3.5 GHz		
**SuMa** [18]	LiDAR-only, surfel-based		1.39	0.34	100	1-core CPU @ 3.5 GHz		
**Group B: DLIO-reported Newer College Short and UCLA Campus A comparisons (DLIO [115])—Supporting Figure 8a,b.**								
**System**	Architecture	Newer College Short ATE RMSE (m)	Newer College mean computation (ms/scan)	UCLA A E2E error (m)	UCLA A runtime (ms/scan)	Completion/failure	Shared hardware/protocol	Comparative observation
**DLO** [51]	LiDAR-only direct	0.4633	48.10	0.022	20.40	Completed	Intel i7-11800H; no additional method-specific change reported.	LiDAR-only direct reference for the within-study comparison.
**CT-ICP** [22]	LiDAR-only continuous-time direct	0.5552	412.27	0.039	351.85	Completed	Intel i7-11800H; voxelization increased and playback slowed to avoid frame drops.	Motion-distortion handling came with substantially higher reported computation.
**LIO-SAM** [13]	Tightly coupled graph-based	0.3957	179.33	0.022	33.21	Completed	Intel i7-11800H; loop closure enabled.	Lower error than the LiDAR-only baselines on Newer College, with higher computation.
**FAST-LIO2** [88]	Tightly coupled filter-based	0.3775	42.86	0.045	15.39	Completed	Intel i7-11800H; online extrinsic estimation disabled.	Strong accuracy-runtime balance in this reporting study, but not the lowest UCLA A error.
**DLIO** [115]	Tightly coupled nonlinear observer	0.3606	35.74	0.011	10.45	Completed	Intel i7-11800H; continuous-time motion correction.	Lowest selected error and low reported computation within these source-conditioned subsets.
**Group C: Selected first-five NCLT results—Supporting Figure 8b. Reported by SR-LIO [110].**								
**System**	Architecture	Dataset/scope	Accuracy metric	Selected result (m)	Runtime	Completion/failure	Shared hardware/protocol	Comparative observation
**LIO-SAM** [13]	Tightly coupled graph-based	First five NCLT sequences	Mean ATE RMSE	3.473	Not reported	4/5; 2012-05-11 failed	Intel i7-12700, 32 GB; author-provided implementations.	Mean excludes one failed sequence and is not equivalent to five-sequence means.
**FAST-LIO2** [88]	Tightly coupled filter-based	First five NCLT sequences	Mean ATE RMSE	2.880	Not reported	5/5	Same platform and reporting study.	Completed all five sequences.
**DLIO** [115]	Tightly coupled nonlinear observer	First five NCLT sequences	Mean ATE RMSE	6.332	Not reported	5/5	Same platform and reporting study.	Mean is strongly influenced by the nclt_4 result.
**iG-LIO** [98]	Tightly coupled MAP optimization	First five NCLT sequences	Mean ATE RMSE	1.978	Not reported	5/5	Same platform and reporting study.	Second-lowest plotted mean in this source-conditioned comparison.
**Point-LIO** [91]	Tightly coupled EKF-based	First five NCLT sequences	Mean ATE RMSE	4.656	Not reported	5/5	Same platform and reporting study.	Completed all five sequences.
**SR-LIO** [110]	Tightly coupled ESIKF-based	First five NCLT sequences	Mean ATE RMSE	1.808	Not reported	5/5	Same platform; NCLT IMU interpolated to 100 Hz.	Lowest plotted mean in the source-conditioned comparison.
**Group D: Selected Robot Town results underlying Figure 8c. Reported by Adaptive-LIO [86].**								
**System**	Architecture	Dataset/scope	Accuracy information (m)	Mean runtime	Completion/failure	Shared hardware/protocol	Comparative observation	
**Adaptive-LIO** [86]	Loosely coupled adaptive	Robot Town Buildings 1–3	1.21/1.36/1.38	31.2 ms/frame	3/3	Livox Avia, RealSense D435i, ROS, 16 GB, “Intel i5-12700H” as reported.	Slower than several baselines but completed all sequences.	
**FAST-LIO2** [88]	Tightly coupled filter-based	Robot Town Buildings 1–3	6.45/16.90/1.94	10.4 ms/frame	3/3	Same reporting study and platform.	Faster than Adaptive-LIO, but accuracy varied strongly across buildings.	
**Point-LIO** [91]	Tightly coupled EKF-based	Robot Town Buildings 1–3	2.38/3.83/1.37	9.9 ms/frame	3/3	Same reporting study and platform.	Fast and completed all three sequences.	
**LIO-SAM** [13]	Tightly coupled graph-based	Robot Town Buildings 1–3	Localization failed on all three	57.5 ms/frame	0/3	Same reporting study and platform.	Reported timing does not indicate successful localization.	
**DLIO** [115]	Tightly coupled nonlinear observer	Robot Town Buildings 1–3	12.38/1.40/1.66	5.9 ms/frame	3/3	Same reporting study and platform.	Low runtime but inconsistent accuracy across buildings.	
**iG-LIO** [98]	Tightly coupled MAP optimization	Robot Town Buildings 1–3	1.32/1.39/Failure	4.9 ms/frame	2/3	Same reporting study and platform.	Fastest reported mean, but one sequence failed.	
